# New theoretical insights into doping-induced enhancement of ORR activity in molybdenum disulfide: d–p hybridization or the Jahn–Teller effect?

**DOI:** 10.1039/d5sc07227a

**Published:** 2026-01-02

**Authors:** Jia-Cheng Chen, Mao-Jun Pei, Wen-Bei Yu, Xiang Gao, Qing Zeng, Jia-Ming Xu, Wei Yan, Yao Liu, Guo-Qiang Luo, Jiujun Zhang

**Affiliations:** a College of Materials Science & Engineering, Fuzhou University Fuzhou 350108 China yaoliu@fzu.edu.cn jiujun.zhang@fzu.edu.cn; b State Key Laboratory of Advanced Technology for Materials Synthesis and Processing, Wuhan University of Technology Wuhan 430070 China luogq@whut.edu.cn

## Abstract

Molybdenum disulfide (MoS_2_) has emerged as a promising electrocatalyst, garnering considerable attention in recent years. However, the extensive basal-plane sites remain intrinsically inert, thereby limiting the overall catalytic efficiency. Heteroatom doping has been demonstrated as an effective strategy for activating these otherwise inert sites; nevertheless, theoretical investigations remain relatively limited, and the broad diversity of dopants has led to conflicting interpretations of the underlying mechanisms. To elucidate the role of dopants in activating these sites, a total of 64 MoS_2_-based electrocatalysts incorporating 3d, 4d, and 5d transition metals, along with selected nonmetals, have been systematically investigated. The results reveal two distinct enhancement pathways: (i) d–p hybridization (d^1^–d^3^ dopants), which elevates the sulfur p-band center and reduces the oxygen reduction reaction (ORR) overpotential to 0.87 V; and (ii) the Jahn–Teller effect (d^7^–d^9^ dopants), which lifts the orbital degeneracy between d_*xz*_/d_*yz*_ and d_*x*^2^−*y*^2^_/d_*xy*_, thereby inducing lattice distortion. The electron rearrangement at the metal center reduces charge transfer, thereby lowering the electron occupancy of the sulfur atom, upshifting its p-band center, and enhancing ORR performance by decreasing the overpotential to 0.53 V. In summary, these findings provide new theoretical insights into substitutional doping and establish guiding principles for the rational design of efficient MoS_2_-based ORR electrocatalysts.

## Abstract 2

Systematic screening reveals two mechanisms, d−p hybridization and Jahn−Teller distortion, that modulate the electronic structure of MoS_2_, enabling substantial ORR overpotential reduction and offering design principles for efficient catalysts.

## Graphical abstract

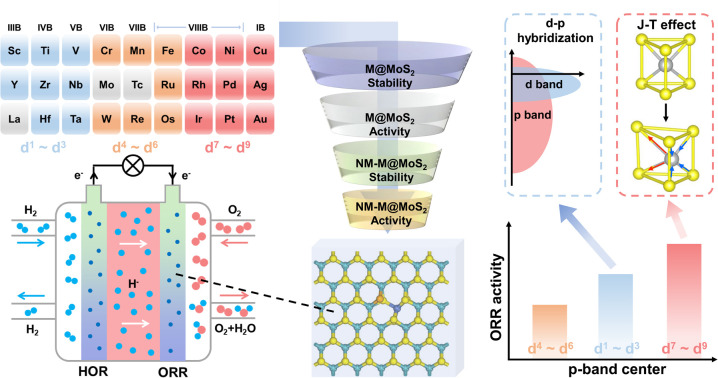

## 1 Introduction

The rapid consumption of fossil fuels to meet the demands of industry development has raised serious concerns regarding resource depletion and environmental degradation, both of which pose a serious threat to the sustainable development of human society.^1^ To mitigate these challenges, the exploration of clean and renewable energy sources, such as solar, wind, and hydropower, together with advances in energy storage and conversion technologies, constitutes a promising avenue toward sustainable energy development.^2,3^ For electrical energy storage and conversion, electrochemical technologies, *e.g.*, water electrolysis for hydrogen production and fuel cells for hydrogen-to-electricity conversion, have been widely recognized as efficient and practical solutions. In particular, fuel cells are distinguished by their high energy efficiency and intrinsic environmental advantages, which have attracted considerable attention over the past decades and position them as highly promising candidates for applications in both transportation and distributed power generation.^4,5^ It is well established that fuel cells operate through two fundamental electrochemical reactions: the hydrogen oxidation reaction (HOR) at the anode and the oxygen reduction reaction (ORR) at the cathode. The ORR, involving multiple intermediate steps in the conversion of O_2_ to H_2_O, is widely regarded as one of the most critical catalytic processes.^6^ Nevertheless, its intrinsically sluggish kinetics, governed by a complex multi-electron transfer pathway, severely hinder overall performance and remain a principal bottleneck to practical implementation.^7^

To date, a diverse array of electrocatalysts have been developed to accelerate the rate of ORR, and many of which have demonstrated notable progress. Among these materials, Pt-based electrocatalysts exhibit outstanding ORR activity and have been successfully commercialized. The prohibitive cost and limited natural abundance of Pt/C electrocatalysts remain formidable obstacles to large-scale deployment, motivating extensive efforts to develop non-precious-metal alternatives.^8,9^ Recently, two-dimensional (2D) materials have attracted widespread attention owing to their unique structural features and tunable physicochemical properties.^10,11^ For example, 2D graphene has been extensively utilized as a support for constructing single-atom ORR electrocatalysts, *e.g.*, Fe–N–C, Co–N–C, and Sn–N–C.^12–16^ Owing to its ultrathin structure, graphene provides shortened charge–transport pathways and excellent electrical conductivity, thereby facilitating electron transfer at metal active sites and enhancing overall catalytic performance. *In situ* characterization techniques have unequivocally confirmed that electrocatalytic reactions, such as the hydrogen evolution reaction (HER), oxygen reduction reaction (ORR), oxygen evolution reaction (OER), carbon dioxide reduction reaction (CO_2_RR), and nitrogen reduction reaction (NRR), primarily occur at metal active sites.^17–20^ Consequently, while M–N–C electrocatalysts can exhibit remarkable catalytic activity, the intrinsic high surface-area advantage of 2D materials remains insufficiently utilized, as the active centers are confined to isolated single atoms.^21^ In this context, 2D materials containing metal atoms, such as layered-double-hydroxides (LDHs), metal sulfides, and MXenes, have attracted considerable attention as another representative developmental pathway, as they inherently provide a greater number of active sites without the need for additional single-atom incorporation.^22–24^ Among these, molybdenum disulfide MoS_2_ has been extensively investigated, primarily due to its availability, chemical stability, and low cost.^25^ Nevertheless, its catalytic activity is largely confined to edge sites, resulting in a limited number of accessible active centers. By contrast, the metal sites on the basal plane are strongly coordinated and therefore remain largely inert, severely restricting the overall catalytic performance of MoS_2_.

To address these limitations, extensive efforts have been devoted to modulating the electronic structure and optimizing the surface properties of MoS_2_ through diverse physical and chemical modifications. Defect engineering, strain modulation, and heteroatom doping have emerged as effective strategies to enhance the intrinsic activity of basal-plane atoms and activate otherwise inert surface regions.^26,27^ For instance, MoS_2_ nanosheets enriched with edge defects was fabricated *via* H_2_O_2_ treatment,^28^ and the optimized electrocatalyst exhibited an onset potential of 0.94 V and a half-wave potential of 0.80 V in 0.1 M KOH. Simultaneously, lanthanide dopants were introduced to modulate the physicochemical properties of MoS_2_.^29^ It revealed that lanthanide doping could modulate the ORR activity of MoS_2_ by altering 4f–5d6s orbital hybridization and Ln–S bonding interactions. In addition, a Co- and Se-codoped MoS_2_ nanofoam with superior catalytic performance was also constructed.^30^ The synergistic interaction between Co and Se not only activated the inner Co sites but also stabilized the surface Se sites, thereby achieving a substantial enhancement in the catalytic performance of MoS_2_. It can be seen that the aforementioned studies clearly demonstrate the critical role of atomic-scale structural regulation in modulating the catalytic behavior of MoS_2_.

At the current state-of-the-art, MoS_2_ is predominantly recognized for its excellent performance in the HER, which has directed the majority of research efforts toward this area. By contrast, investigations into its ORR activity remain relatively limited, largely owing to the prevailing assumption that the active sites are Mo atoms, which are typically highly coordinated and therefore less catalytically active. Specifically, the substantial steric hindrance and high coordination stability of Mo atoms on the basal plane render them unsuitable for binding oxygen-containing intermediates, thereby constraining the practical applicability of MoS_2_ as an ORR electrocatalyst. Fortunately, the recent study has demonstrated that rational heteroatom doping can effectively reconfigure the active sites from Mo to S atoms, thereby enhancing the electrocatalytic ORR activity of MoS_2_.^29^ This approach provides a promising strategy for re-engineering MoS_2_ as an ORR electrocatalyst by fully leveraging its abundant intrinsic active sites. Despite recent advances in experiments, theoretical investigations remain limited, and the wide diversity of dopants has often led to conflicting interpretations of the underlying mechanisms. In particular, the effects of transition metal doping on the ORR mechanism, such as its influence on orbital hybridization, charge transfer, and intermediate adsorption at sulfur sites, have not been systematically explored. This has resulted in a critical gap in understanding the structure–activity relationships that underpin the rational design of high-performance ORR electrocatalysts; hence, further theoretical investigations are both necessary and timely.

Accordingly, a series of MoS_2_-based electrocatalysts doped with various transition-metal (M@MoS_2_, M = metal) and non-metal elements (NM–M@MoS_2_, NM = non-metal) are constructed in this study to systematically investigate how different dopants modulate ORR catalytic performance. Based on the calculated overpotentials and the degree of lattice distortion in M@MoS_2_ before and after structural relaxation, the doped transition metals can be classified into three categories according to the number of outermost d-orbital electrons, *i.e.*, d^1^–d^3^, d^4^–d^6^, and d^7^–d^9^. The enhanced ORR performance of M@MoS_2_ is primarily attributed to the upward shift of the sulfur p-band center, which optimizes the adsorption strength of ORR intermediates and lowers the associated reaction energy barriers, thereby improving the overall catalytic activity. However, the underlying mechanisms responsible for this shift vary across the different classes of dopants discussed above. For transition metals in the d^1^–d^3^ region, the sulfur p-band center (3p) is modulated by the dopant d-band electrons (3d, 4d, and 5d) through a d–p orbital hybridization effect. In contrast, doping elements with electronic configurations in the d^7^–d^9^ range induce significant structural distortion in M@MoS_2_. Therefore, the upshift of the p-band center is attributed to the Jahn–Teller effect. Under this circumstance, the degeneracy between the d_*xz*_/d_*yz*_ and d_*x*^2^−*y*^2^_/d_*xy*_ orbitals is lifted, leading to a rearrangement of the electronic configuration at the metal center to maintain structural stability. A reduced number of electrons are transferred to the sulfur atom, thereby lowering its electron occupancy and elevating the p-band center, which effectively activates the sulfur active site, as evidenced by a reduced overpotential in the range of 0.53–0.74 V. Building on these findings, five non-metal elements are selected for dual-doping to further improve the catalytic activity, *e.g.*, C–, N–, O–, P–, and Se–M@MoS_2_. Among them, only the systems incorporating Se as the non-metal dopant displayed outstanding ORR activity, achieving overpotentials in the range of 0.41–0.50 V. In summary, this study presents a comprehensive theoretical framework for synergistically tuning the electronic structure of MoS_2_-based electrocatalysts through metal and non-metal co-doping, thereby providing new avenues for the rational design of highly selective and efficient ORR electrocatalysts.

## 2 Computational details

In this study, first-principles calculations were performed within the framework of density functional theory (DFT) using the Vienna *Ab initio* Simulation Package (VASP).^31–33^ The exchange–correlation interactions were described using the generalized gradient approximation (GGA) with the Perdew–Burke–Ernzerhof (PBE) functional.^34^ The ion–electron interactions were conducted using the projector-augmented wave (PAW) approach, with a plane-wave energy cutoff of 450 eV.^35,36^ The exposed (001) facet of MoS_2_, which is of greater interest, was therefore selected as the focus of this investigation. A vacuum layer of 15 Å was introduced along the *z*-direction to effectively eliminate spurious interactions between periodic images. To evaluate the reliability of the computational methodology, comparative calculations were performed using 3 × 3 and 4 × 4 supercells, respectively, *i.e.*, Co@MoS_2_, Ni@MoS_2_, and Cu@MoS_2_. As shown in Fig. S1 of the SI, only minor discrepancies in the overpotentials are observed between these results, *i.e.*, 0.58 V (0.61 V), 0.53 V (0.53 V), and 0.65 V (0.67 V). Therefore, a 3 × 3 supercell was adopted for geometry optimization and static calculations to conserve computational resources.

The Brillouin zone was sampled with a 3 × 3 × 1 *k*-point mesh for geometry optimizations and a 12 × 12 × 1 mesh for electronic structure computations. Notably, all atomic positions were fully relaxed without any spatial constraints. Spin-polarized calculations were employed, and long-range van der Waals interactions were incorporated using the DFT-D3 dispersion correction method within Grimme's scheme to improve computational accuracy.^37,38^ The convergence thresholds for atomic force and electronic energy were set to 0.01 eV Å^−1^ and 10^−5^ eV, respectively, during the structural optimization. Atomic charge analysis was conducted using the atom-in-molecule (AIM) approach as proposed by Bader.^39^ To enable a more detailed investigation of interatomic bonding interactions, Crystal Orbital Hamilton Population (COHP) analysis was carried out using the LOBSTER 5.0.0 package.^40,41^*Ab initio* molecular dynamic (AIMD) simulations were performed in the NVT ensemble at 500 K, with the system temperature controlled by the Nosé–Hoover thermostat.^42^ A time step of 1 fs was employed, and the simulation was conducted for 10 ps to evaluate the structural and dynamic stability. The VASPKIT code was utilized for post-processing the computational data obtained from VASP.^43,44^ Migration barriers were determined using the climbing-image nudged elastic band (CI-NEB) method.^45^ The O_2_ dissociation barrier was computed by constrained AIMD combined with the slow-growth protocol, employing a collective-variable increment of 4 × 10^−4^ Å.^46,47^ In addition, implicit solvent effects were evaluated for pristine MoS_2_ with VASPsol.^48,49^ As shown in Fig. S2, the ORR overpotential differs by only 0.09 V between vacuum and implicit solvent conditions, indicating a minimal solvent correction. Accordingly, unless otherwise noted, all remaining calculations were performed under vacuum conditions. The formation energy (*E*_for_) quantifies the thermodynamic cost of incorporating dopants into the host lattices and serves as an indicator of system stability, which can be expressed as

1 *E*_for_ = *E*_tot_ − *E*_sub_ − (*E*_M_ + *E*_NM_)

*E*_tot_ and *E*_sub_ represent the total energies of MoS_2_ with and without dopants, respectively. *E*_M_ represents the total energy of the transition metal dopant in its most stable bulk phase, while *E*_NM_ corresponds to the total energy of the non-metal dopant in its most stable form. The dissolution potential (*U*_diss_) refers to the electrochemical potential at which a material or alloy begins to dissolve in an electrolyte under specific conditions. It is defined as:

2 *U*_diss_ = *U*^*Θ*^_diss(metal,bulk)_ − *E*_for_/*eN*_e_

where *U*^*Θ*^_diss(metal,bulk)_ is the standard dissolution potential of bulk metal and *N*_e_ is the number of electrons in solution, and can be obtained from the ref. 50. According to the above definitions, a negative formation energy indicates that doping is thermodynamically favorable, whereas a positive dissolution potential suggests that the metal atoms in the electrocatalysts are resistant to dissolution under electrochemical conditions. Moreover, the associative four-electron pathway of the oxygen reduction reaction (ORR) is outlined below^51^

3 O_2_ + * + H^+^ + e^−^ → OOH*

4 OOH* + H^+^ + e^−^ → O* + H_2_O(l)

5 O* + H^+^ + e^−^ → OH*

6 OH* + H^+^ + e^−^ → * + H_2_O(l)

* signifies the active site, while OH*, O*, and OOH* denote the adsorbed oxygen-containing intermediates. To evaluate the catalytic activity, the Gibbs free energy change (Δ*G*_*i*_, where *i* = 1, 2, 3, 4) for each step of the ORR process is calculated using the computational hydrogen electrode (CHE) model

7 Δ*G*_ads_ = Δ*E* + ΔZPE − *T*Δ*S* − *neU* − *k*_b_*T*  ln[H^+^]

here, Δ*E* denotes the reaction energy, ΔZPE represents the zero-point energy difference. The term *T*Δ*S* accounts for the entropy contribution, where Δ*S* is the entropy difference and *T* is the Kelvin temperature, set at 298.15 K. *eU* denotes the potential and *n* is the number of transferred charges. *k*_b_*T* ln[H^+^] = −*k*_b_*T* ln 10 × pH, which represents the correction to the free energy of H^+^ due to its concentration, where *k*_b_ is the Boltzmann constant. Notably, a zero voltage under the acidic condition is adopted in this work, *i.e.*, *U* = 0 and pH = 0. The overpotential, *i.e.*, *η*, which stands for the ORR activity can be defined as:

8 Δ*G*_min_ = min{Δ*G*_1_, Δ*G*_2_, Δ*G*_3_, Δ*G*_4_}

9 *η* = 1.23 − Δ*G*_min_/*e*

It is evident that a lower overpotential indicates a reduced energy barrier, underscoring the minimization of *η* as a crucial factor in the design of high-performance electrocatalysts. Based on eqn (8) and (9), it can be inferred that achieving the minimum *η* necessitates the Gibbs free energy of the four fundamental steps to be identical, specifically 1.23 eV.^52^

## 3 Results and discussion

### 3.1 Structural and thermodynamic stability

**Fig. 1:**
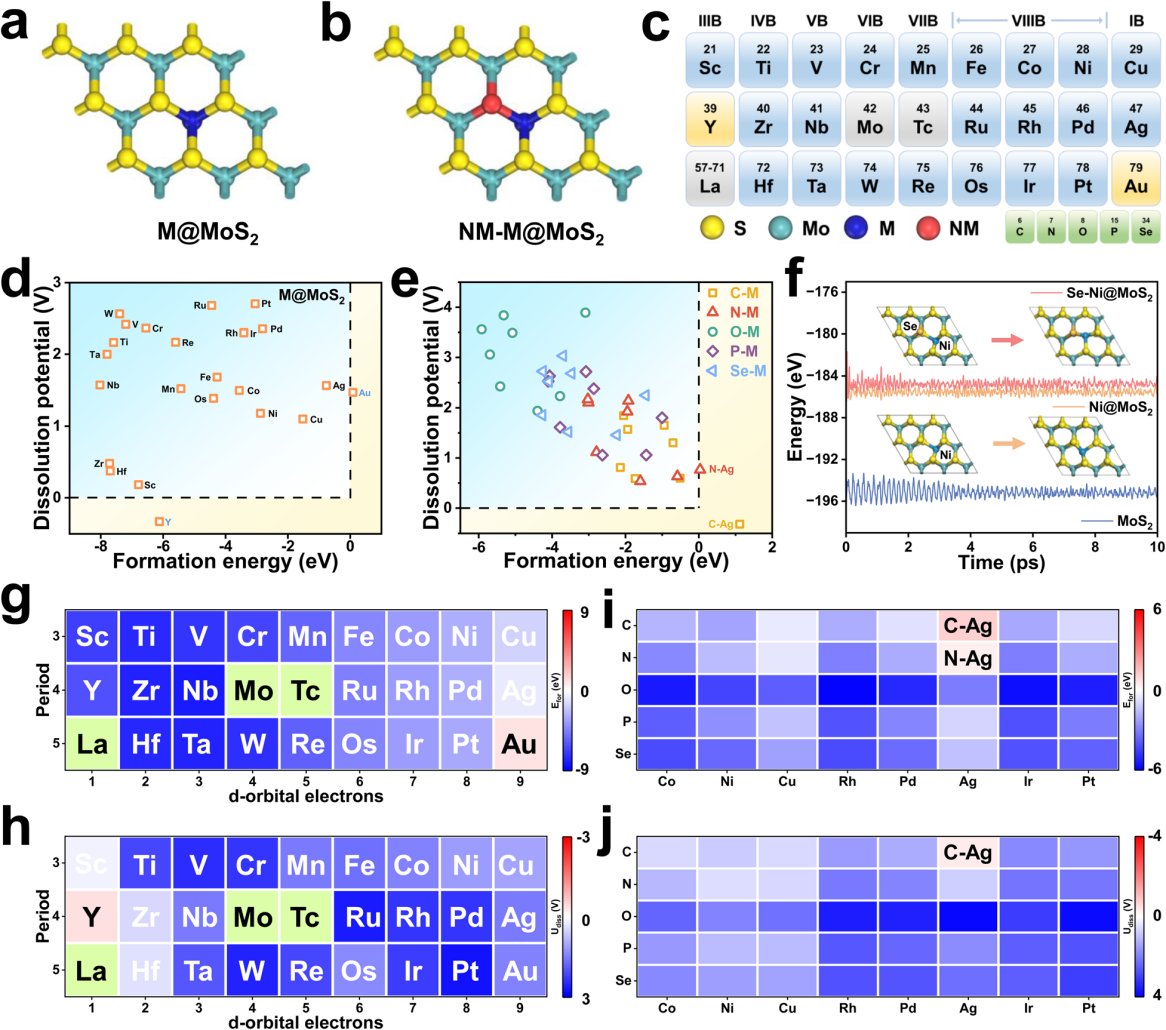
(a) and (b) Structural models of M@MoS_2_ and NM–M@MoS_2_. (c) Schematic representation of the 24 transition-metal dopants and 5 non-metal dopants. (d) and (e) Screening of structural and thermodynamic stability for M@MoS_2_ and NM–M@MoS_2_, respectively. (f) Energy evolution profiles over a 10 ps AIMD simulation for MoS_2_, Ni@MoS_2_, and Se–Ni@MoS_2_, along with the initial and final structures of Ni@MoS_2_, and Se–Ni@MoS_2_. (g)–(j) Calculated formation energies and dissolution potentials for M@MoS_2_ and NM–M@MoS_2_.

Fig. 1a and b depict schematic illustrations of M@MoS_2_ and NM–M@MoS_2_, where “M” and “NM” denote the doped metal and non-metal elements, respectively. To identify the preferred doping site of transition metals on MoS_2_, the formation energies of four representative configurations are calculated, *e.g.*, Mo_top_, Vs_top_, S_sub_, and Mo_sub_, as shown in Fig. S3. Ni@MoS_2_ is selected as a representative example, with the corresponding formation energies are 1.21, 1.73, 0.32, and −2.88 eV, respectively. Therefore, Mo_sub_ is identified as the most thermodynamically stable doping position, and subsequent investigations will focus on this configuration.^29,53^Fig. 1c presents a total of 24 dopants for MoS_2_, spanning the 3d, 4d, and 5d transition metal series, where technetium (Tc) and the lanthanides are excluded due to concerns regarding toxicity and radioactivity. To eliminate the influence of outermost orbitals, *i.e.*, 4s, 5s, and 6s, the transition metals are classified by disregarding two valence electrons. Accordingly, “M” can be classified as d^1^ to d^9^, depending on the number of d-orbital electrons in their penultimate or outermost shell.

Structural and thermodynamic stability are essential criteria for evaluating the viability of the constructed electrocatalysts, *e.g.*, M@MoS_2_ and NM–M@MoS_2_. Consequently, their formation energies and the dissolution potentials are calculated and presented in Fig. 1d and e, respectively, with detailed values provided in Table S1. Fig. 1d and g show that the formation energies for single-atom doping are negative in all cases except Au@MoS_2_, *i.e.*, *E*_for_ = 0.09 eV, indicating that most M@MoS_2_ structures are thermodynamically stable. In addition, Fig. 1h illustrates that all constructed M@MoS_2_ systems exhibit positive dissolution potential values, except for Y@MoS_2_, which presents a dissolution potential of −0.33 eV. Based on the aforementioned results, a total of 22 stable M@MoS_2_ electrocatalysts are selected for subsequent investigations, excluding Y@MoS_2_ and Au@MoS_2_. To quantify the degree of structural distortion, the stability is examined before and after geometry relaxation, with the distortion index (*D*) defined as follows:10
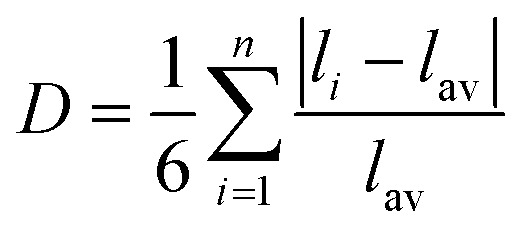
here, *n* represents the coordination number of the metal atom, *l*_*i*_ denotes the length of each individual bond, and *l*_av_ is the average bond length.^54^ Fig. S4 summarizes the distortion indices of M@MoS_2_, showing that dopants in the d^1^–d^6^ range induce only minimal or negligible structural distortions, thereby preserving structural integrity and maintaining the original coordination environment. In contrast, the incorporation of d^7^–d^9^ transition metals result in distortion indices ranging from 4 × 10^−2^ to 12 × 10^−2^, consistent with previous studies.^55^

In view of the growing interest in leveraging diatomic synergistic effects to develop high-performance electrocatalysts, a portion of NM–M@MoS_2_ samples are investigated and the schematics are shown in Fig. S5. As the following results demonstrate that M@MoS_2_ doped with d^7^–d^9^ transition metals can exhibit superior ORR performance, thus are selected as the “M” component in NM–M@MoS_2_, *i.e.*, M = Co, Ni, Cu, Rh, Pd, Ag, Ir, Pt, and Au. Five non-metal elements (NM = C, N, O, P, and Se) are chosen for co-doping, yielding a total of 40 NM–M@MoS_2_ electrocatalysts. Fig. S6 depicts the dual-doped configuration exhibit structural distortions comparable to those observed in the single-doped system, *i.e.*, 0.12 (Ni@MoS_2_) and 0.13 (Se–Ni@MoS_2_). Consequently, the structural distortion of NM–M@MoS_2_ is primarily attributed to the transition metal dopants rather than the non-metal dopants. As shown in Fig. 1e, i and j, the calculated formation energies and the dissolution potentials of NM–M@MoS_2_ are presented. Except for C–Ag@MoS_2_ and N–Ag@MoS_2_, which exhibit the formation energies of 1.12 and 0.04 eV, respectively, the remaining 38 configurations demonstrate satisfactory stability and are suitable for analysis. To further assess the kinetic stability, dopant migration barriers at the Mo sites were calculated for Ti@MoS_2_, Cr@MoS_2_, and Ni@MoS_2_. As shown in Fig. S7, the barriers are 11.12, 10.36, and 4.62 eV for Ti, Cr, and Ni, respectively, indicating strong dopant–lattice interactions and thus negligible diffusion. In addition, AIMD simulations were carried out to monitor their structural evolution over time. Fig. 1f shows that the total energies remain stable throughout the 10 ps simulation, and only negligible structural deformation can be observed. Se–Ni@MoS_2_ and Ni@MoS_2_ exhibit higher total energies compared to pristine MoS_2_, *i.e.*, −184.79, −185.54, and −195.16 eV, further indicating that the introduction of dopants disrupts the structure. Such lattice destabilization can reduce reaction barriers and enhance catalytic activity, similar to the effects observed in amorphous structures, thereby highlighting the potential of these doped configurations as efficient ORR electrocatalysts.

### 3.2 Mechanism of the ORR activity

#### 3.2.1 ORR activity of M@MoS_2_

At the current state-of-the-art, Mo atoms situated at the edges, rather than within the bulk, are widely recognized as the active sites in MoS_2_-based electrocatalysts, as shown in Fig. S8. This is attributed to their lower coordination environment, which typically results in superior catalytic activity.^56^ In addition, S atoms can effectively adsorb reaction intermediates and actively participate in the catalytic process; therefore, they are also considered active centers.^29,57^ In this study, the (001) surface of MoS_2_ is investigated, where Mo atoms reside within a stable trigonal prismatic coordination environment, conferring both high structural stability and pronounced steric hindrance, as show in Fig. 5b. To verify this conclusion, calculations reveal that OH* species initially adsorbed on Mo atoms spontaneously migrate to adjacent S atoms upon structural relaxation in Ti@MoS_2_, Cr@MoS_2_, and Ni@MoS_2_, indicating that OH* adsorption on Ni sites is strongly hindered by substantial steric effects, as illustrated in Fig. S9. Fig. S10 presents the adsorption energies of OH* at various sites, *i.e.*, S_1_ to S_7_, revealing that S_5_ exhibits the highest adsorption energy, *i.e.*, −2.62 eV. Consequently, the surface S atoms, particularly those adjacent to the dopant, are more likely to function as catalytically active sites than the bulk Mo atoms.

To further address potential ambiguities in the identification of active sites, ORR free energy profiles were calculated for both edge Mo and edge S sites on the MoS_2_ (100) surface, as shown in Fig. S11a and c. The results indicate that the ORR overpotential at the edge Mo site is 2.87 V, substantially higher than that at the S site on the (001) surface, *i.e.*, 1.93 V. Fig. S11b and d reveal that the edge Mo site binds ORR intermediates much more strongly than the surface S site; for instance, the adsorption free energy of *OH is approximately 1.94 eV at the S site on the (001) surface but around −2.13 eV on the Mo-edge site. In contrast, the ORR overpotential at the edge S site is only 0.78 V, suggesting that edge S sites are more likely to function as the actual highly active catalytic centers. However, previous studies have demonstrated that MoS_2_ edges exhibit poor chemical stability and are prone to oxidation and corrosion under electrochemical conditions,^58^ which undermines the practical viability of edge S sites as stable catalytic centers. Accordingly, S sites on the (001) surface are selected as the active centers for systematic investigation, both to elucidate the impact of electronic-structure modulation and to reflect realistic operational constraints.^59,60^

**Fig. 2:**
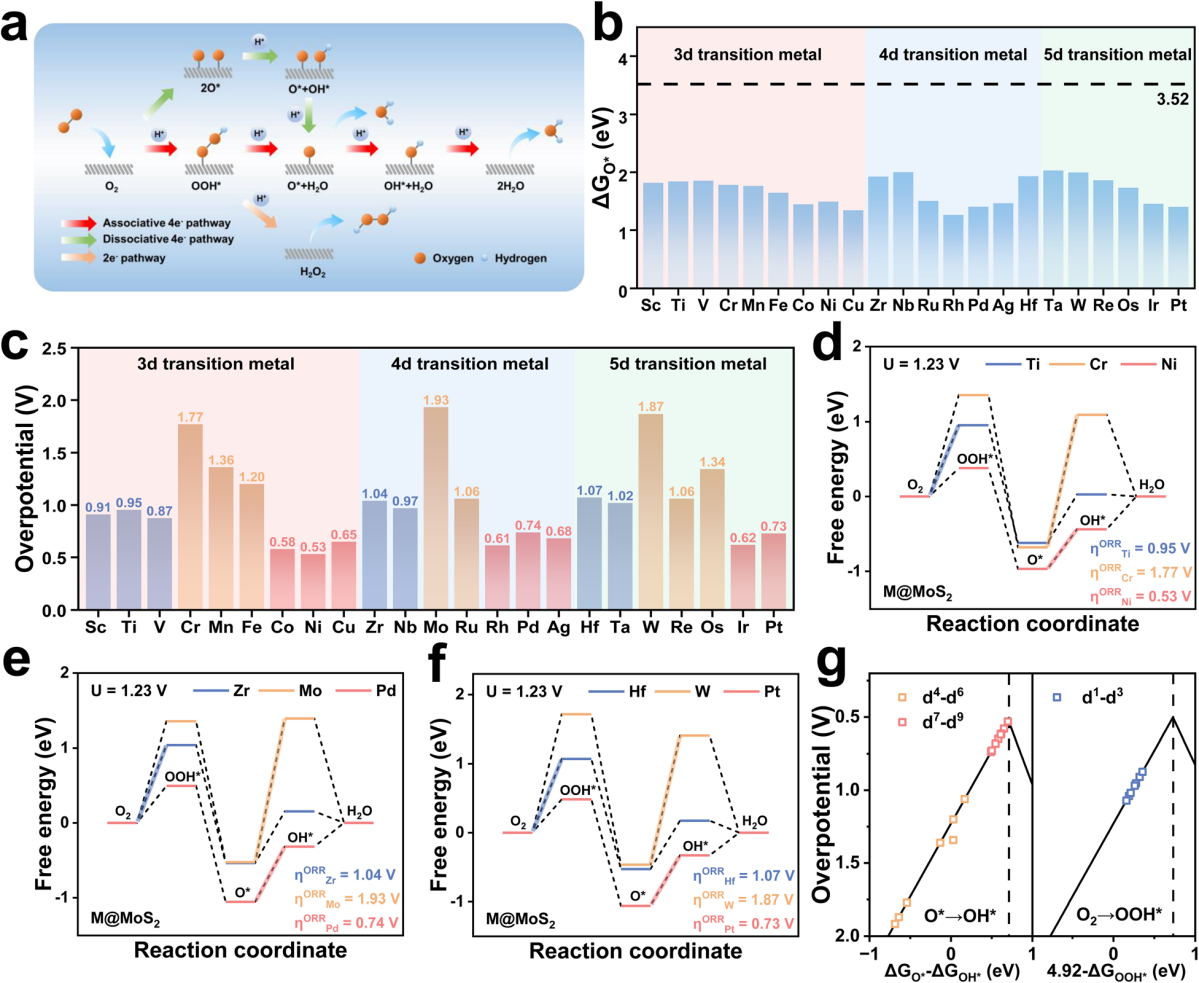
(a) Schematic illustration of the oxygen reduction reaction (ORR) pathways. (b) Statistical comparison of Δ*G*_O*_ values for 22 M@MoS_2_ configurations. (c) Calculated overpotentials (*η*) of the electrocatalysts. Free energy diagrams for representative doped M@MoS_2_ samples: (d) Ti, Cr, and Ni; (e) Zr, Mo, and Pd; (f) Hf, W, and Pt. (g) Volcano plot of ORR activity for M@MoS_2_.

Fig. 2a illustrates three possible pathways may occur during the ORR. According to previous studies,^61^ the dissociation activation barrier of O_2_ on MoS_2_-based electrocatalysts is approximately 1.59 eV, rendering the dissociative pathway kinetically unfavorable. In view of the fact that doping and lattice distortion may significantly influence the O_2_ dissociation pathway, the energy barrier was further evaluated for a representative system in the d^7^–d^9^ region, *i.e.*, Ni@MoS_2_. Constrained AIMD combined with a slow-growth scheme was employed to probe O_2_ dissociation, with the O–O bond length serving as the collective variable for direct evaluation of the finite-temperature free-energy barrier (Δ*E*_free_). As shown in Fig. S12, the O_2_ dissociation barrier on Ni@MoS_2_ is 0.62 eV, substantially lower than that on pristine MoS_2_*i.e.*, 3.10 eV, indicating that lattice distortion indeed facilitates O–O bond cleavage. Nevertheless, it is noteworthy that O–O bond cleavage with an activation barrier exceeding 0.6 eV is kinetically hindered at practical electrode potentials.^62^ Under realistic conditions, Ni@MoS_2_ is expected to favor the associative pathway as the dominant ORR mechanism rather than the dissociative pathway.

To initiate the ORR process, O_2_ should first be adsorbed onto the catalyst surface. Fig. S13 presents the calculated adsorption energies of O_2_ on the surfaces of M@MoS_2_, which range from −0.28 to −0.06 eV. Experimental evidence suggests that the adsorption of O_2_ proceeds *via* a two-step process: an initial reduction of O_2_ to OOH^−^ in the outer Helmholtz plane, followed by the adsorption of OOH^−^ onto the electrocatalyst surface to form OOH*.^63^ Although the activation barrier for the O_2_ → OOH^−^ conversion is relatively low, the ORR is not significantly affected by the weak O_2_ adsorption energy. After OOH* adsorbs on the surface of M@MoS_2_, two competing routes may proceed: (i) a four-electron pathway that generates water, *i.e.*, OOH* + H^+^ + e^−^ → O* + H_2_O, or (ii) a two-electron pathway that yields hydrogen peroxide, *i.e.*, OOH* + H^+^ + e^−^ → H_2_O_2_. As shown in Fig. S14, it is widely acknowledged that when Δ*G*_O*_ is below 3.52 eV, the electrocatalyst exhibits strong O* adsorption capability, thereby facilitating the associative four-electron ORR pathway for H_2_O production.^64^ On this basis, Fig. 2b presents the Gibbs free energy of O*, showing that all M@MoS_2_ systems exhibit values below 3.52 eV. Therefore, the associative four-electron pathway is thermodynamically favored and will therefore serve as the primary focus of the main text.

Fig. 2c and e show that the calculated ORR overpotential of MoS_2_ is 1.93 V, and the potential-determining step (PDS) is the protonation of O* to OH*. Compared with MoS_2_, transition-metal doping enhances the catalytic activity of M@MoS_2_ to varying extents, with the overpotentials ranging from 0.53 to 1.87 eV. Moreover, the overpotential initially increases with the rising d-electron count and subsequently decreases, exhibiting a periodic trend across the different main groups. Taking the 3d transition metals as examples, *e.g.*, Sc, Ti, V, Cr, Mn, Fe, Co, Ni, and Cu, the corresponding overpotentials are 0.91, 0.95, 0.87, 1.77, 1.36, 1.20, 0.58, 0.53, and 0.65 V, respectively. Based on these findings, the dopants can be roughly categorized into three categories according to their d-electron count, *i.e.*, d^1^–d^3^, d^4^–d^6^, and d^7^–d^9^. For transition metals with electronic configurations in the d^7^–d^9^ range, the corresponding M@MoS_2_ exhibits superior activity compared to other dopants. The overpotentials follow the order: Ni (0.53 V) < Co (0.58 V) < Rh (0.61 V) < Ir (0.62 V) < Cu (0.65 V) < Ag (0.68 V) < Pt (0.73 V) < Pd (0.74 V). In contrast, doping with transition metals in the d^1^–d^3^ range results in moderate enhancement relative to MoS_2_, with the overpotential remaining around 1 V. The introduction of d^4^–d^6^ metals offers limited improvement, as most M@MoS_2_ exhibit overpotentials exceeding 1.2 V. In addition, Fig. 2c and Table S2 reveal an intriguing phenomenon that doping with Cr (3d^4^) or W (5d^4^), metals whose outermost d-orbital electronic configurations closely resemble that of Mo (4d^4^), yields overpotentials for Cr@MoS_2_ and W@MoS_2_ that are comparable to MoS_2_, with values of 1.77, 1.87, and 1.93 V, respectively. Fig. 2d to f display the free-energy diagrams of representative electrocatalysts from the three activity regions, including Ti (3d^2^), Cr (3d^4^), Ni (3d^8^), Zr (4d^2^), Mo (4d^4^), Pd (4d^8^), Hf (5d^2^), W (5d^4^), and Pt (5d^8^). The overpotential of M@MoS_2_ initially increases and then decreases, with the corresponding values are 0.95 (Ti), 1.77 (Zr), and 0.53 V (Ni), in the case of 3d transition metal dopants. A similar trend is also observed in the 4d (Zr, Mo, and Pd) and 5d (Hf, W, and Pt) transition metal series, and the free-energy diagrams for the remaining electrocatalysts are provided in Fig. S15.

Fig. 2g illustrates the overpotential volcano plot encompassing all M@MoS_2_, aiming to identify the factor governing catalytic activity. It is evident that for transition metals with d^4^–d^9^ configurations, the PDS corresponds to Δ*G*_3_ (O* → OH*), whereas Δ*G*_1_ (O_2_ → OOH*) is identified as the PDS for those in the d^1^–d^3^ region. In addition, M@MoS_2_ doped with d^7^–d^9^ metals cluster to the left of the vertex, indicating strong OH* adsorption, whereas those doped with d^4^–d^6^ transition metals lie farther from the vertex, suggesting weaker OH* adsorption.^65^ For M@MoS_2_ doped with d^1^–d^3^ transition metals, moderate OH* adsorption is observed relative to the other two groups, and the PDS is identified as OOH* adsorption. These findings underscore the critical role of OH* adsorption in dictating the catalytic performance of M@MoS_2_-based electrocatalysts. Accordingly, the subsequent discussion adopts OH* as the primary descriptor, providing a unified framework for interpreting adsorption trends across various metal dopants.

#### 3.2.2 P-band center

**Fig. 3:**
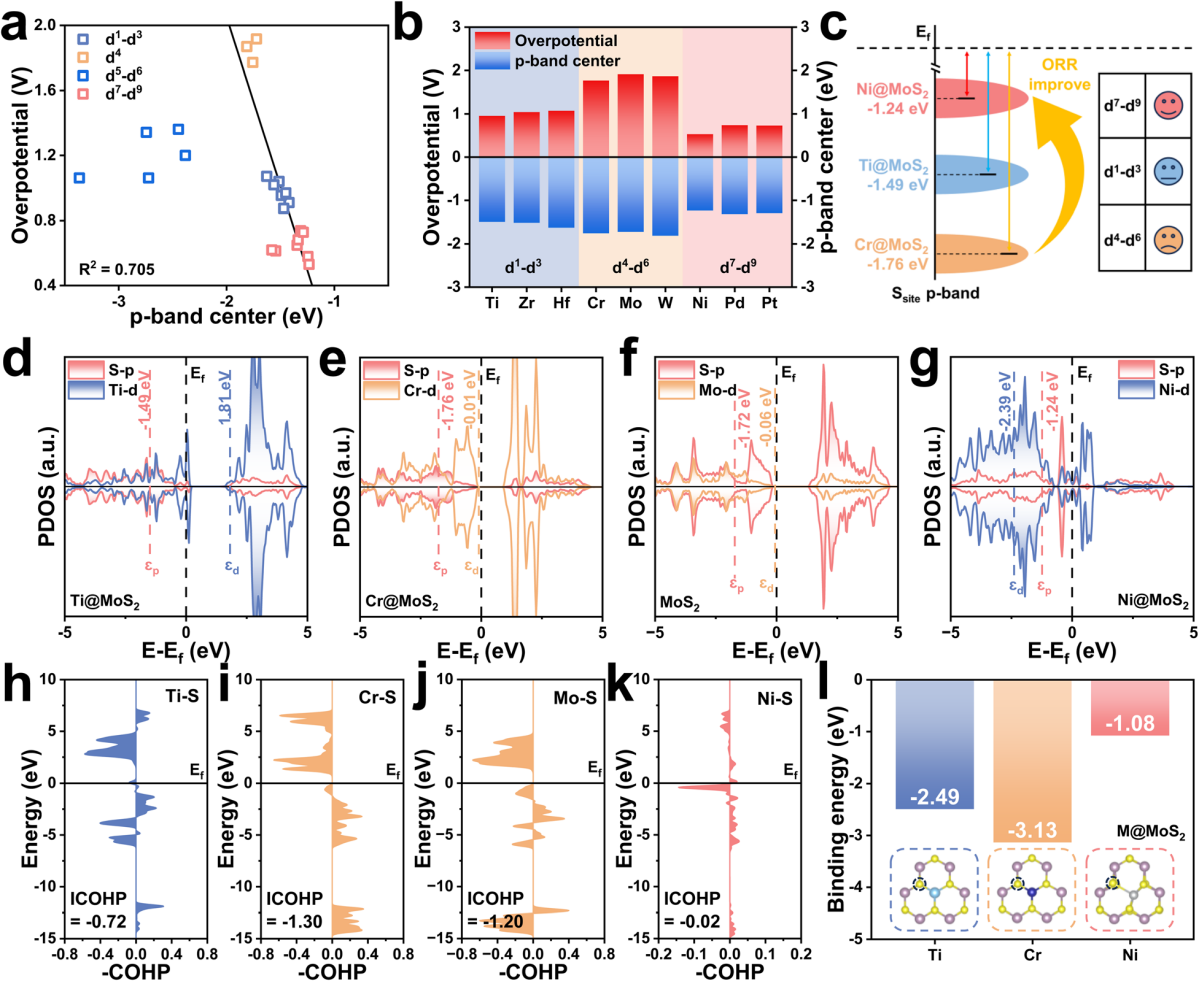
(a) Scaling relationship between the overpotential (*η*) and the sulfur p-band center (*ε*_p_) of M@MoS_2_. (b) Correlation of the overpotential and the p-band center for MoS_2_ and M@MoS_2_ as a function of the nominal d-orbital electron count. (c) Schematic illustration of the relationship between *η* and *ε*_p_. Projected density of states (PDOS) of the metal d-orbitals and the sulfur p-orbitals for (d) Ti@MoS_2_, (e) Cr@MoS_2_, (f) MoS_2_, and (g) Ni@MoS_2_. (h)–(k) Crystal orbital Hamilton population (COHP) analyses of the M–S bonds for Ti@MoS_2_, Cr@MoS_2_, MoS_2_, and Ni@MoS_2_, respectively. (l) Corresponding binding energies of these three electrocatalysts.

The ORR performance is closely linked to electron transfer at the active centers and the magnitude of their valence-state changes. Consequently, both the p-band center (*ε*_p_) and the d-band center (*ε*_d_) are employed as descriptors of the electrocatalytic activity.^66,67^ As the preceding discussion established the surface S_5_ site as the active center, the underlying mechanism can be elucidated by analyzing its p-band center. Fig. 3a illustrates a linear correlation between *ε*_p_ and *η*, yielding a coefficient of determination *R*^2^ = 0.705, excluding the cases of MoS_2_ doped with d^5^–d^6^ transition metals. Fig. 3b depicts several representative M@MoS_2_ with dopants in different main groups, and detailed values are provided in Table S3. Transition metals with analogous outermost d-orbital electronic configurations exhibit comparable enhancements in catalytic activity, as evidenced by the overpotentials of 0.95 V for Ti@MoS_2_ (3d^2^), 1.04 V for Zr@MoS_2_ (4d^2^), and 1.07 V for Hf@MoS_2_ (5d^2^). The corresponding *ε*_p_ values are −1.49, −1.51, and −1.63 eV, respectively, indicating a linear relationship between catalytic performance and the position of the p-band center. As illustrated in Fig. 3c, the proposed mechanism underscores that the p-band center position at the active site governs its interaction with oxygen-containing intermediates, thereby modulating the overall activity of M@MoS_2_.

Ti@MoS_2_, Cr@MoS_2_, and Ni@MoS_2_, which belong to the same period and represent the d^1^–d^3^, d^4^–d^6^, and d^7^–d^9^ categories, respectively, are selected for detailed investigation. As shown in Fig. S4, Ti@MoS_2_ and Cr@MoS_2_ exhibit no structural distortions upon doping, indicating that the observed activity differences are attributed primarily to variations in *ε*_p_. To elucidate the influence of electronic configuration on catalytic activity and modulation of the p-band center, Fig. 3d to g present the PDOS of Ti@MoS_2_, Cr@MoS_2_, MoS_2_ and Ni@MoS_2_. For Ti@MoS_2_, the p-band center of the active S-atom and the d-band center of the dopant Ti-atom are −1.49 and 1.81 eV, respectively, whereas for Cr@MoS_2_ these values shift to −1.76 and −0.01 eV. By comparison, pristine MoS_2_ exhibits corresponding values of −1.72 and −0.06 eV, while in Ni@MoS_2_, the p- and d-band centers are positioned at −1.24 and −2.39 eV, respectively. Additionally, the PDOS reveals that the overlapping region between Cr and S locates at a lower energy compared to that between Ti and S, indicative of a stronger Cr–S bond. This is corroborated by the ICOHP values for the Ti–S, Cr–S, Mo–S, and Ni–S bonds presented in Fig. 3h–k, *e.g.*, −0.72, −1.30, −1.20, and −0.02. Fig. 3l further shows that the binding energies of the S-atom adjacent to the dopant are −2.49, −3.13, and −1.08 eV, respectively. These findings collectively suggest that d electrons of the M-atom transfer to the neighboring S-atom, thereby modulating its p-band center, which can be referred to as the d–p orbital hybridization effect. Specifically, Cr and Mo possess identical outermost d-orbital electronic configurations, and their corresponding *ε*_d_ values are closely aligned, *i.e.*, −0.01 and −0.06. This suggests that the degree of d–p orbital hybridization, and consequently its effect on the p-band center, should be comparable in Cr@MoS_2_ and pristine MoS_2_. Consistent with this expectation, the corresponding values of *ε*_p_ for these two electrocatalysts are nearly identical, *i.e.*, −1.76 and −1.72 eV. Due to the much higher *ε*_d_ of Ti compared with Cr, *i.e.*, 1.81 and −0.01 eV, the *ε*_p_ of the active S atom in Ti@MoS_2_ shifts upward significantly under strong d–p hybridization. In contrast, Ni@MoS_2_ doping induces pronounced structural distortion, which weakens the Ni–S bond interaction, *i.e.*, ICOHP = −0.02. Therefore, the upward shift of *ε*_p_ in Ni@MoS_2_ is more reasonably ascribed to distortion effects rather than d–p hybridization.^68^

**Fig. 4:**
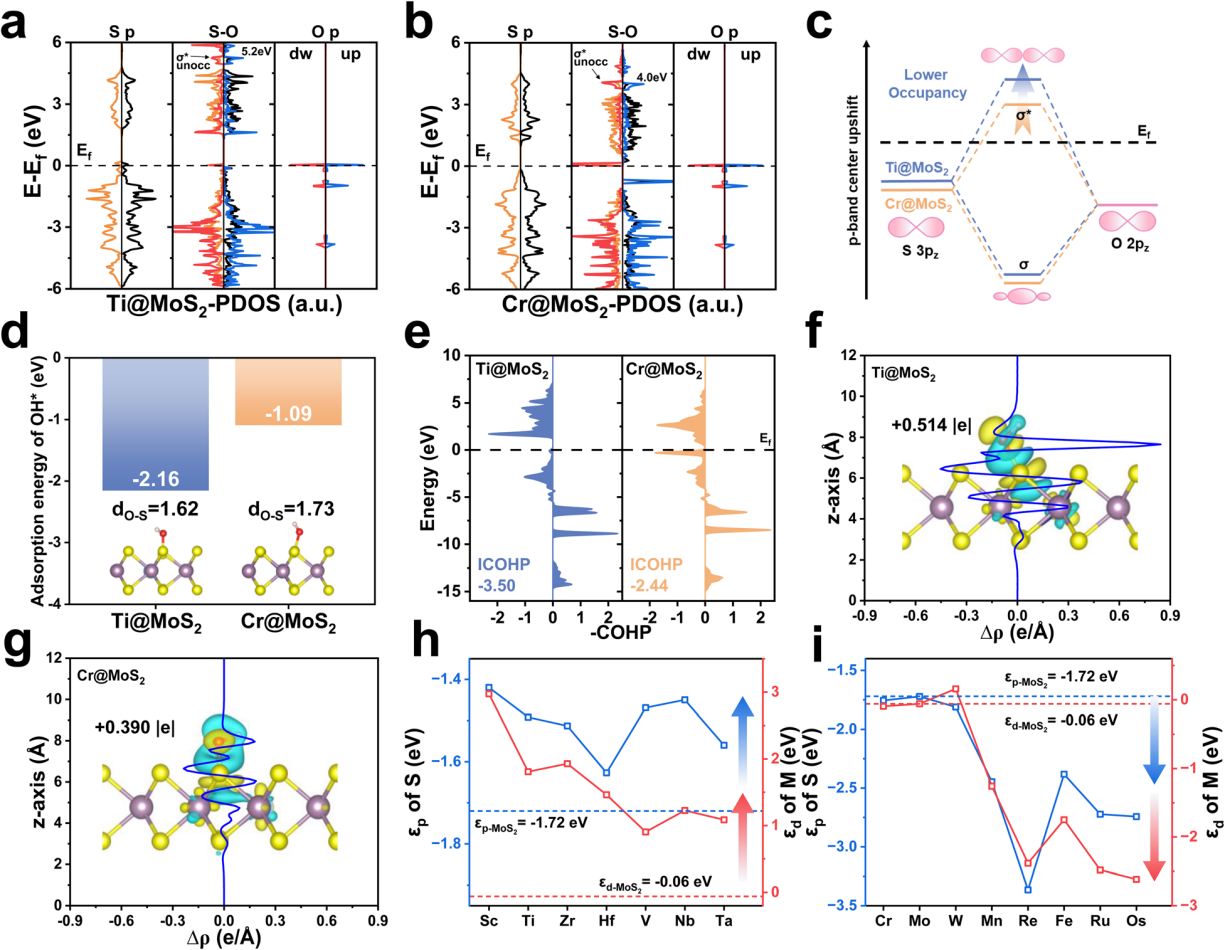
(a) and (b) PDOS of isolated sulfur and oxygen atoms for Ti@MoS_2_ and Cr@MoS_2_, along with their interaction following OH* adsorption. (c) Schematic illustration of the effect of Ti doping on the bonding interaction between O and S atoms. (d) Adsorption energies of OH* on Ti@MoS_2_ and Cr@MoS_2_. (e) Corresponding COHP analyses of O–S bonds in Ti@MoS_2_ and Cr@MoS_2_. (f) and (g) Plane-averaged charge density difference *ρ*(*z*) and associated charge transfer from S to OH* for Ti@MoS_2_ and Cr@MoS_2_. (h) and (i) *ε*_d_ of the metal dopant and *ε*_p_ of the sulfur for MoS_2_ doped with transition metals in the d^1^–d^3^ and d^4^–d^6^ categories, respectively.

Fig. 4a and b present the PDOS for OH* adsorption on Ti@MoS_2_ and Cr@MoS_2_, respectively. The left and right panels display the active S-atom and the O-atom of OH*, respectively, both of which are in their isolated states, and the selected atoms are shown in Fig. S16. It reveals that the O 2p orbitals partially overlaps with the S 3p orbitals, indicating the bonding of S–O. The corresponding PDOS of the two systems during S–O interaction is presented in the center, with spin-up and spin-down states distinguished by different colors. Fig. 4c presents a molecular-orbital schematic illustrating the interaction between O and S atoms upon OH* adsorption, which results in the formation of σ bonding (O 2p–S 3p) and σ* (O 2p–S 3p) antibonding orbitals, respectively. Due to the aforementioned d–p hybridization effect, Ti doping elevates the energy level of the S 3p orbitals, resulting in an upward shift of the p-band center in Ti@MoS_2_ relative to Cr@MoS_2_. The resulting σ* antibonding orbital is also elevated, accompanied by a decrease in the occupation of the O–H σ* orbital, forming a more robust S–O bond, which implies an increase in the adsorption of OH*. As shown in Fig. 4d, the adsorption energy of OH* on Ti@MoS_2_ (−2.16 eV) is substantially more negative than that on Cr@MoS_2_ (−1.09 eV), indicating stronger adsorption. Correspondingly, the S–O bond lengths are shortened to 1.62 and 1.73 Å, respectively, further indicating that OH* binding on Ti@MoS_2_ is more thermodynamically favorable. In addition, Fig. 4e demonstrates that the ICOHP value of the S–O bond in Ti@MoS_2_ is −3.50, lower than the value of −2.44 in Cr@MoS_2_, validating the aforementioned analysis. As shown in Fig. 4f and g, the Bader charge and the plane-averaged charge density difference reveal the charge transfer characteristics of M@MoS_2_ toward the adsorbed OH*, providing insights into the charge distribution. Regarding Ti@MoS_2_, the O-atom of OH* accepts 0.514 |e|, which is significantly higher than 0.390 |e| observed in Cr@MoS_2_. This enhancement is attributed to the upward shift of the σ* antibonding orbitals associated with OH* adsorption induced by Ti doping, which reduces its electronic occupancy and thereby strengthens the S–O bond.

The preceding discussion demonstrates that the improved ORR performance of M@MoS_2_, particularly for dopants with d^1^–d^3^ configurations, arises from modulation of the p-band center through the d–p hybridization effect, *i.e.*, M (3d, 4d or 5d)–S (3p). Fig. 4h and i summarize the *ε*_d_ values of all d^1^–d^6^ dopants and the *ε*_p_ values of their corresponding active sites to illustrate the d–p hybridization effect. It can be seen that the similar trends of the *ε*_d_ and *ε*_p_ curves underscore a clear linear correlation, highlighting the pronounced influence of the d–p hybridization effect. As shown in Fig. 4h, the *ε*_d_ values of these transition metals are higher than that of Mo when the dopants are located in d^1^–d^3^, and the resulting d–p hybridization leads to an upward shift in the p-band centers of the active sites upon doping. Fig. 4i depicts that d^4^–d^6^ transition metals exhibit the *ε*_d_ values comparable to or even lower than that of Mo. As a result, the d–p hybridization effect yields *ε*_p_ that are also comparable to or lower than that of Mo. Nevertheless, the electrocatalytic activities of Fe@MoS_2_, Ru@MoS_2_, and Os@MoS_2_ are superior to that of MoS_2_, despite their lower p-band centers. These results appear to contradict the aforementioned conclusion that *ε*_p_ closer to the Fermi level can improve the catalytic activity. This phenomenon is attributable to the structural distortion introduced by d^4^–d^6^ dopants in M@MoS_2_. For transition metals with electronic configurations ranging from d^1^–d^3^, the relaxed M@MoS_2_ structures exhibit no significant structural distortion. Accordingly, the p-band center in these systems is modulated solely by the d–p interaction, accounting for the observed enhancement in catalytic performance. By contrast, with d^4^–d^6^ and d^7^–d^9^ configurations, both the d–p hybridization effect and the lattice distortion should be considered, as the latter can introduce additional complexity and new mechanism. Hence, the d–p hybridization effect alone cannot explain the behavior across all M@MoS_2_ cases.

#### 3.2.3 Jahn–Teller effect

**Fig. 5:**
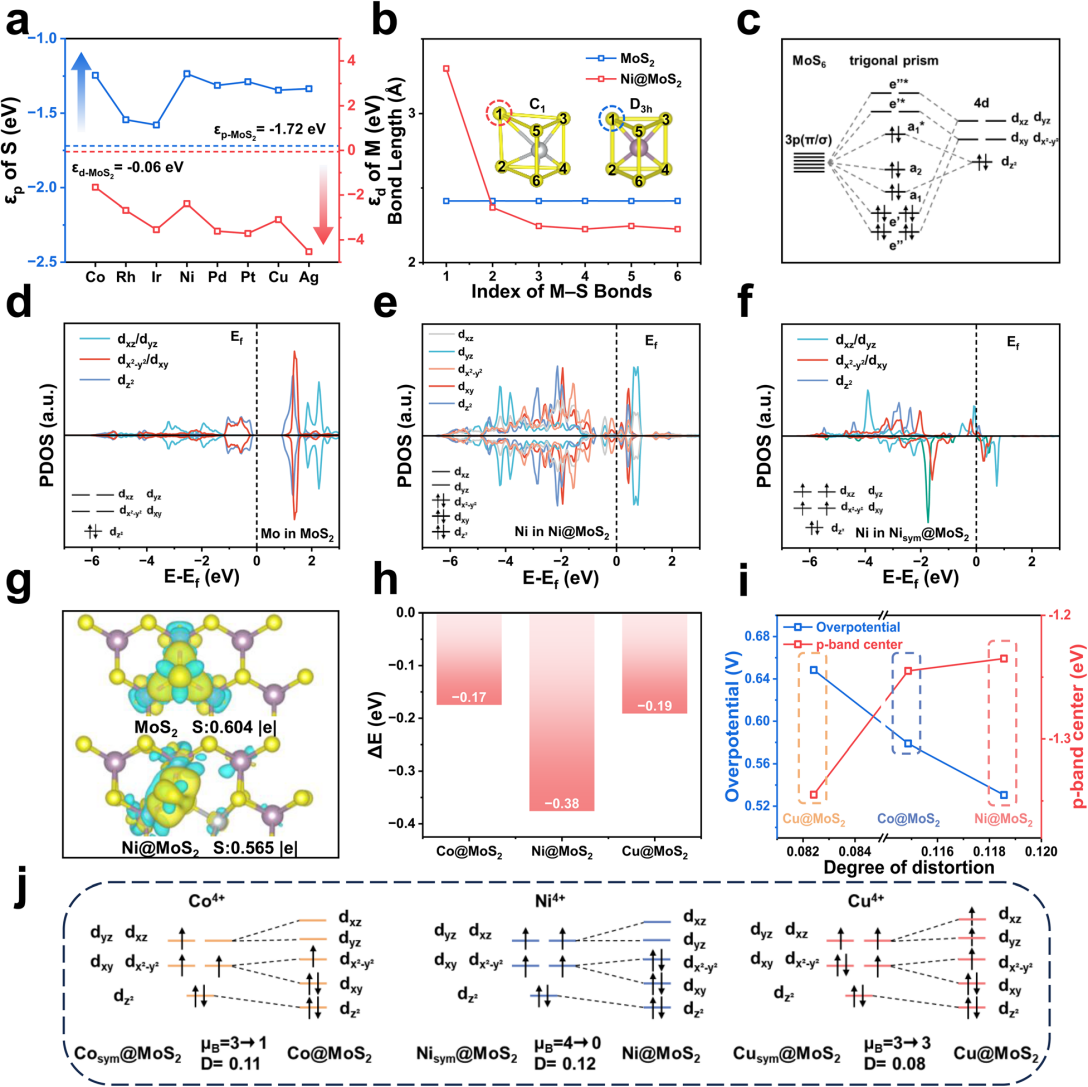
(a) Energy levels of the metal dopant d-orbitals (*ε*_d_) and the sulfur p-orbitals (*ε*_p_) in M@MoS_2_ doped with d^7^–d^9^ transition metals. (b) Bond lengths of representative M–S bonds in pristine MoS_2_ and Ni@MoS_2_. (c) Schematic illustration of orbital energy-level splitting in MoS_2_. PDOS of d-orbitals for (d) Mo in pristine MoS_2_, (e) Ni in Ni@MoS_2_, and (f) Ni in symmetric Ni_sym_@MoS_2_. (g) Charge density difference and Bader charge analysis for MoS_2_ and Ni@MoS_2_. (h) Energy variations before and after structural distortion for Co@MoS_2_, Ni@MoS_2_, and Cu@MoS_2_. (i) Correlation between the degree of structural distortion, the sulfur p-band center (*ε*_p_), and the ORR overpotential. (j) Orbital distribution diagrams of Co@MoS_2_, Ni@MoS_2_, and Cu@MoS_2_ before and after distortion.

Doping elements with electronic configurations in the d^7^–d^9^ range can induce significant structural distortion in M@MoS_2_, and the resulting effects can no longer be neglected. As shown in Fig. 5a, the d-band center of Ni@MoS_2_ is −2.39 eV, which is lower than that of pristine MoS_2_, *i.e.*, −0.06 eV. Fig. S17 illustrates that the p-band center of the active S-atom to −1.24 eV with the introduction of Ni-atom, compared to −1.72 eV in MoS_2_. The p-band center shifts upward, contrary to the expected downward shift associated with the d–p hybridization effect, thereby deviating from the anticipated behavior. The similar upshifts in the p-band center are observed across the d^7^–d^9^ range, indicating that this phenomenon is not unique but rather reflects a systematic trend, as shown in Fig. 5a. Compared to Fig. 4h and i, the elevated p-band center is unlikely to result from the d–p hybridization effect, but is more likely attributable to lattice distortion, suggesting a distinct underlying mechanism.

To elucidate this effect, MoS_2_ and Ni@MoS_2_ are discussed in Fig. 5b, revealing significant differences in their geometric configurations. All Mo–S bond lengths in MoS_2_ are approximately 2.4 Å, indicating that Mo atoms are situated in a regular trigonal prismatic coordination environment with D3h symmetry. Consequently, MoS_2_ exhibits a high degree of d-orbital degeneracy, with notable overlap between the d_*xz*_/d_*yz*_ and d_*xy*_/d_*x*^2^−*y*^2^_ orbitals,^23^ as shown in Fig. 5d. With the introduction of Ni-atom, Ni@MoS_2_ exhibits a distinctly coordination environment with *C*1 symmetry, with one bond significantly elongated to 3.3 Å and the remaining bonds shortened to 2.2–2.3 Å. Fig. 5e depicts a pronounced splitting of the d orbitals in Ni@MoS_2_, including d_*xz*_, d_*yz*_, d_*xy*_, d_*x*^2^−*y*^2^_, d_*z*^2^_. To further confirm that the orbital splitting arises from lattice distortion rather than differences in the metal center, *i.e.*, Mo and Ni. Ni_sym_@MoS_2_ with D3h symmetric and no lattice distortion has been constructed as a comparative reference. Fig. 5f shows that, even for the Ni atom, the same orbital degeneracies observed in MoS_2_ are retained, specifically d_*xz*_/d_*yz*_ and d_*xy*_/d_*x*^2^−*y*^2^_. These results demonstrate that the disruption of orbital degeneracy is not dependent on the dopant element but is closely associated with the coordination environment. This phenomenon, known as the Jahn–Teller effect, induces orbital energy level splitting and modulates the electronic configuration, thereby playing a critical role in governing ORR performance. Further calculations on MoS_2_ and Ni@MoS_2_ are presented to verify the role of the Jahn–Teller effect in modulating the electronic configuration. Fig. S18 illustrates that the ICOHP value of the M–S bond increases from −1.20 to −0.02, indicating a substantial weakening of the bonding interaction due to the Jahn–Teller effect. Additionally, the S-atom receives 0.604 |e| from the metal center, which decreases to 0.565 |e| in Ni@MoS_2_, indicating reduced electron transfer upon Ni doping, as shown in Fig. 5g. The reduced charge transfer lowers the electron occupancy of the S-atom 3p orbitals, thereby elevating the 3p-band center and strengthening the S–OH* coupling. To validate this conclusion, Fig. S19 shows that the electron transfer from the S-atom to OH* are 0.340 |e| and 0.444 |e| for the Ni-doped and undoped cases, respectively. These results not only demonstrate that the electronic structure of the S-atom is closely associated with the Jahn–Teller effect, but also provide direct evidence of an intrinsic correlation between the p-band center and enhanced adsorption capacity.

To deepen the understanding, a detailed molecular-orbital analysis is subsequently performed to elucidate how the Jahn–Teller effect modulates the electronic configuration of the active center. Fig. 5c depicts the molecular orbitals of the MoS_6_ cluster, comprising a series of bonding, antibonding, and non-bonding orbitals.^69^ From the perspective of classical chemistry theory, the valence state of Mo is designated as +4, thus Mo^4+^ is adopted for the electronic structure analysis. According to the reference, the six S atoms contribute twelve lone pairs that occupy the low-energy bonding orbitals, *i.e.*, e″, e′, and a_1_, as well as the non-bonding orbital, *i.e.*, a_2_. The remaining high-energy antibonding orbitals are governed chiefly by the d-electrons of Mo, *i.e.*, d^2^, with two d electrons occupying the 
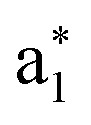
 antibonding orbital.^70^ The metal center in the cluster will be substituted with various transition metal dopants, *e.g.*, M@MoS_2_. When the number of d-orbital electrons is low, *i.e.*, d^1^–d^3^, fewer electrons occupy the 
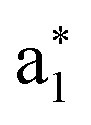
 antibonding orbital, resulting in a relatively stable MS_6_ cluster that retains the original trigonal prismatic coordination structure. As the d-electron count increases to d^5^ and d^6^, electrons begin to occupy the higher-energy e′* antibonding orbitals, destabilizing the system and inducing slight distortions in the coordination environment. With a further increases to d^7^–d^9^, electrons may occupy the highest-energy e″* antibonding orbital, rendering in pronounced instability, as shown in Fig. S4. At this stage, the Jahn–Teller effect reduces orbital degeneracy through structural distortion, thereby lowering the total energy and enhancing structural stability. Fig. 5h illustrates that the total energy of Ni_sym_@MoS_2_ is significantly higher than that of Ni@MoS_2_, *i.e.*, −190.81 and −191.19 eV, corresponding to a difference of −0.38 eV. To further validate this trend, the energy differences between undistorted and distorted configurations are calculated for Co (Co_sym_@MoS_2_/Co@MoS_2_) and Cu (Cu_sym_@MoS_2_/Cu@MoS_2_) transition metal dopants, yielding values of −0.17 and −0.19 eV, respectively. It can be seen that the distorted structures consistently display lower energies, indicating that structural distortion is thermodynamically favorable and contributes to system stabilization. To validate the change in orbital degeneracy, the magnetic moments of Co, Ni, and Cu in M_sym_@MoS_2_ are calculated to analyze their electron configurations. As shown in Fig. 5j, *µ*_B_ = 3, 4, and 3 for the corresponding M_sym_@MoS_2_ systems, in which the cluster retains a regular trigonal prismatic coordination environment. Moreover, the degenerate orbitals can be clearly observed, *i.e.*, d_*xz*_/d_*yz*_ and d_*xy*_/d_*x*^2^−*y*^2^_. Under the realistic conditions, accounting for the Jahn–Teller effect, the magnetic moments of M@MoS_2_ decrease to 1, 0, and 3, respectively. Consequently, the orbital degeneracy is lifted, and the electronic configuration adjusts to preserve structural stability. The relationships among structural distortion, the p-band center, and catalytic performance have been investigated, as shown in Fig. 5i. As structural distortion increases, from Cu@MoS_2_ to Co@MoS_2_ and finally to Ni@MoS_2_, the p-band center of the S-atom progressively shifts upward toward the Fermi level. This modulation enhances the adsorption capacity of the active site for ORR intermediates, thereby effectively promoting catalytic performance.

To verify the generality of conclusions, the analysis was expanded from 3d dopants, *e.g.*, Cu, Co, and Ni, to representative 4d and 5d dopants, *e.g.*, Pd, Rh, Ag, Pt, and Ir. As shown in Fig. S20a and b, increasing lattice distortion leads to a pronounced upward shift of *ε*_p_, accompanied by a significant decrease in *η*. This trend is preserved across elements from the 3d, 4d, and 5d series, thereby confirming the generality of the correlation between Jahn–Teller distortion, electronic structure, and catalytic activity. It is worth noting that a few outliers may arise from the omission of explicit relativistic effects (spin–orbit coupling) for the 4d and 5d elements;^71,72^ however, these deviations do not alter the overall trend or the main conclusions. In addition, the edge S site of the MoS_2_ (100) surface was carried out for a comparative analysis under an identical computational framework and convergence criteria. Within the edge environment, Fig. S21a and b depict the ORR activity and p-band center after doping. The results show that, for the MoS_2_ (100) edge systems, the overpotential remains strongly correlated with the p-band center (*R*^2^ = 0.89), consistent with the trend established for the (001) surface. The specific values of edge S sites are *η*_MoS_2__ = 0.78 V (*ε*_p_ = −1.37 eV), *η*_Ni@MoS_2__ = 1.04 V (*ε*_p_ = −2.20 eV), and *η*_Ti@MoS_2__ = 1.60 V (*ε*_p_ = −2.73 eV), respectively. In contrast to the doped systems, the pristine MoS_2_ edge S site exhibits superior ORR performance, indicating that introducing dopants at the MoS_2_ (100) edge may suppress rather than enhance the catalytic activity. As shown in Fig. S21c and d, no appreciable d–p hybridization is observed at the edge S sites of Ti@MoS_2_. Regarding the edge S sites in Ni@MoS_2_, although a Jahn–Teller distortion occurs, it does not lead to any enhancement in activity. These results demonstrate that the p-band center activity descriptor established for the (001) surface remains valid at the edge; however, the specific promotion mechanisms identified for the (001) surface cannot be directly transferred to the MoS_2_ (100) edge.

In summary, unlike d^1^–d^3^ transition-metal dopants, d^7^–d^9^ dopants can trigger the Jahn–Teller effect, thereby inducing structural distortion. Moreover, the Jahn–Teller effect produces synergistic consequences, rearranging the orbital energy levels of the dopants and redistributing charge between M–S, that collectively drive a pronounced upward shift of the p-band center of the active S-atom, thereby enhancing its electronic coupling with and adsorption of ORR intermediates. This mechanism not only reveals the deep physical origin of the improved catalytic performance of Ni@MoS_2_, but also provides theoretical support for the construction of transition metal regulation strategies. Fig. S22 further presents the PDOS of nine transition-metal dopants, *i.e.* Sc to Cu. Except for the d^4^ system, all dopants convert MoS_2_ from a semiconductor to a metal, imparting pronounced conductivity. This electronic-structure transformation is expected to enhance electron-transfer efficiency during electrocatalysis, thereby promoting the ORR.

### 3.3 ORR performance with diatomic doping

**Fig. 6:**
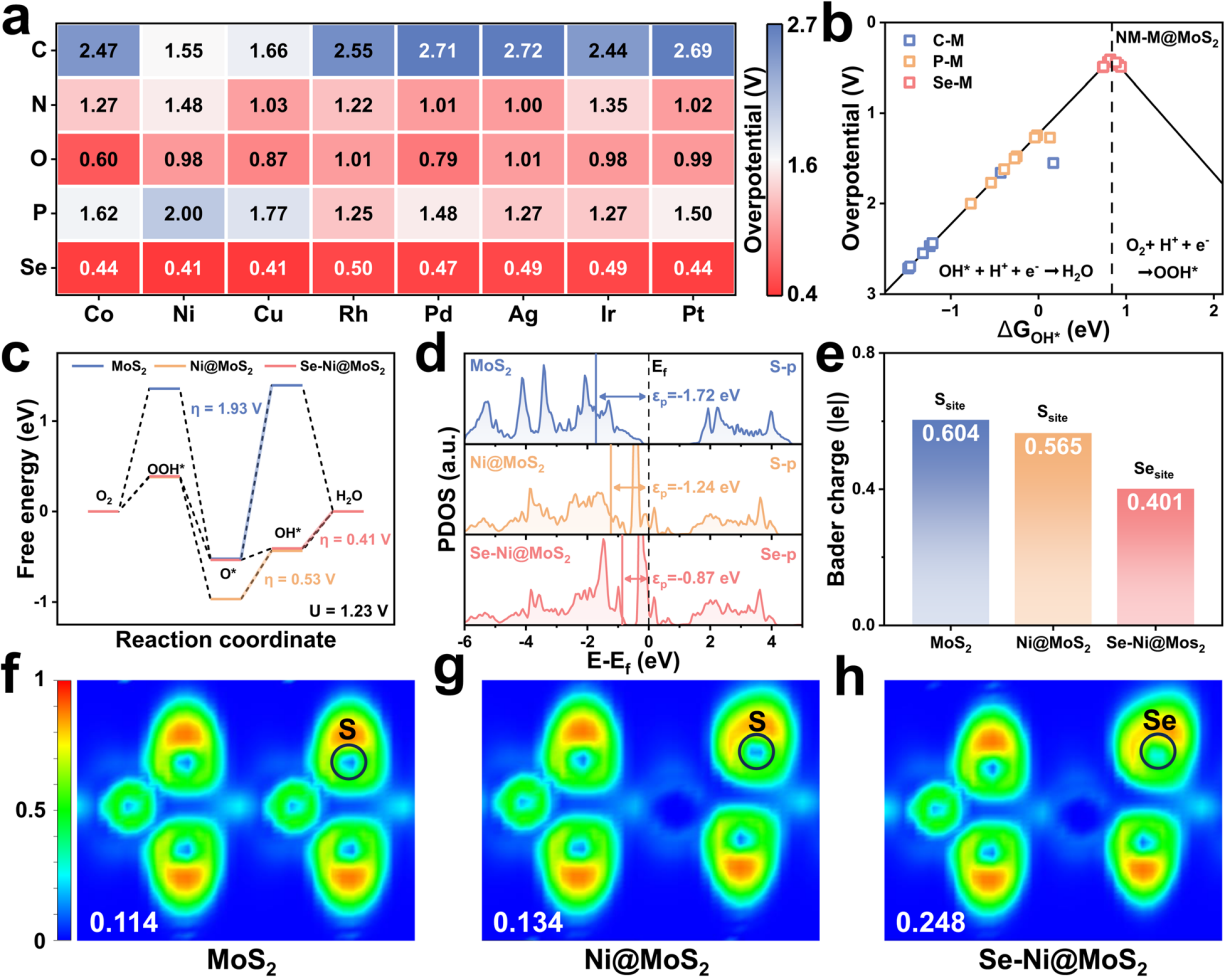
(a) Calculated overpotentials for C, N, O, P, and Se as non-metal dopants in NM–M@MoS_2_. (b) Volcano plot of NM–M@MoS_2_ with C, P, and Se as representative dopants. (c) Free energy diagrams for MoS_2_, Ni@MoS_2_ and Se–Ni@MoS_2_, respectively. (d) PDOS of the 3p orbitals for the active sulfur atom. (e) Charge transfer values from surrounding atoms to the active site in MoS_2_, Ni@MoS_2_ and Se–Ni@MoS_2_. (f)–(h) Corresponding electron localization function (ELF) plots.

As demonstrated in Fig. 2c, M@MoS_2_ doped with transition metals possessing d^7^–d^9^ electronic configurations exhibit superior ORR performance. Consequently, five non-metal elements, *i.e.*, NM = C, N, O, P, and Se, located near sulfur in the periodic table, are selected for diatomic doping with the aim of further enhancing the catalytic activity. Fig. 6a presents the calculated overpotentials of 40 NM–M@MoS_2_ in the form of a heat map. The results indicate that only dual-doped systems incorporating Se as the non-metal dopant exhibit outstanding ORR catalytic activity. Specifically, the overpotentials are ranked as follows: Se–Ni@MoS_2_ (0.41 V) = Se–Cu@MoS_2_ (0.41 V) < Se–Co@MoS_2_ (0.44 V) = Se–Pt@MoS_2_ (0.44 V) < Se–Pd@MoS_2_ (0.47 V) < Se–Ir@MoS_2_ (0.49 V) = Se–Ag@MoS_2_ (0.49 V) < Se–Rh@MoS_2_ (0.50 V). In contrast, NM–M@MoS_2_ incorporating other non-metal elements, such as C, N, O, and P, exhibit comparatively lower catalytic activity. Among these, C–M@MoS_2_ exhibits the largest overpotential, *i.e.*, *η* > 1.6 V, primarily due to its excessively strong adsorption capacity, which signifies substantial reaction barriers during the catalytic process. As shown in Fig. S23, in the case of O–M@MoS_2_, most O* and OOH* adsorption configurations are unstable, indicating structural instability and disqualifying these systems as viable electrocatalyst candidates.

Table S2 indicates that the PDS is primarily Δ*G*_4_ for NM–M@MoS_2_ with C, P, and Se as non-metal dopants. Consequently, Fig. 6b depicts the volcano plot of NM–M@MoS_2_ (NM = C, P, and Se), elucidating the trends in their catalytic activity. It is evident that the Se–metal dual-doped structures exhibit the highest catalytic performance, with Se–Ni and Se–Cu doped MoS_2_ achieving overpotentials of 0.41 V, positioning them near the volcano apex. In contrast, the C–metal and P–metal doped systems exhibit significantly higher overpotentials, indicating that their catalytic activities are constrained by the excessively strong adsorption of OH*. Additionally, the volcano plot of the O–metal and N–metal doped electrocatalysts is presented in Fig. S24 due to their distinct PDS. To elucidate the electronic and structural role of Se in nonmetal co-doping, PDOS, ICOHP and charge density difference analyses for the representative Se–Ni@MoS_2_ and O–Ni@MoS_2_ were shown in Fig. S25. The p-band center of Se–Ni@MoS_2_ is *ε*_p_ = −0.87 eV, whereas that of O–Ni@MoS_2_ is *ε*_p_ = −3.07 eV. A higher *ε*_p_ supports more moderate adsorption of OOH*/OH*, whereas an overly negative *ε*_p_ corresponds to excessively weak adsorption, thereby accounting for the inferior activity of the O co-doped system. The Ni–O bond is markedly stronger than the Ni–Se bond, *i.e.*, ICOHP = −0.49 *vs.* −0.02, indicating that the stronger Ni–O interaction is accompanied by more substantial electron transfer and occupation rearrangement. In contrast, the weaker Ni–Se interaction preserves a higher p-band center and optimal adsorption strengths, consistent with the lower overpotential of Se–Ni@MoS_2_. These results indicate that Se co-doping primarily serves to maintain an elevated p-band center and near-optimal adsorption of oxygenated intermediates, whereas O co-doping induces an excessive downward shift of the p-band center that suppresses ORR activity.

To further investigate the impact of diatomic doping on catalytic activity, MoS_2_, Ni@MoS_2_, and Se–Ni@MoS_2_ are selected as representative models for detailed analysis. The corresponding free energy diagrams and the p-band centers are presented in Fig. 6c and d, respectively. The results indicate that dopants induce a progressive upward shift in the p-band center of the catalyst, *i.e.*, *ε*_p_ = −1.72, −1.24, and −0.87 eV, thereby enhancing its catalytic activity as reflected by the corresponding overpotentials of, *i.e.*, *η* = 1.93, 0.53, and 0.41 V. Fig. 6e and S26 illustrate the calculated Bader charge analysis and the charge density difference, and in pristine MoS_2_, the Mo atom transfers 0.604 |e| to the adjacent S-atom. For comparison, the Ni-atom transfers 0.565 |e| to the active S-atom in Ni@MoS_2_, whereas in Se–Ni@MoS_2_, the electron transfer from Ni to Se decreases to 0.401 |e|. Notably, the reduced electron transfer in Se–Ni@MoS_2_ relative to Ni@MoS_2_ leads to an upward shift of the p-band center. As shown in Fig. 6f to h, the electron localization functions (ELF) of the corresponding electrocatalysts are calculated to further validate these findings. The results reveal a progressive increase in ELF values at the active sites upon Ni and Se–Ni doping, rising from 0.114 to 0.134 and 0.248, respectively. This trend indicates enhanced electron localization around the active sites and a corresponding reduction in electron transfer to the surrounding atoms, consistent with the Bader charge analysis.^73^

**Fig. 7:**
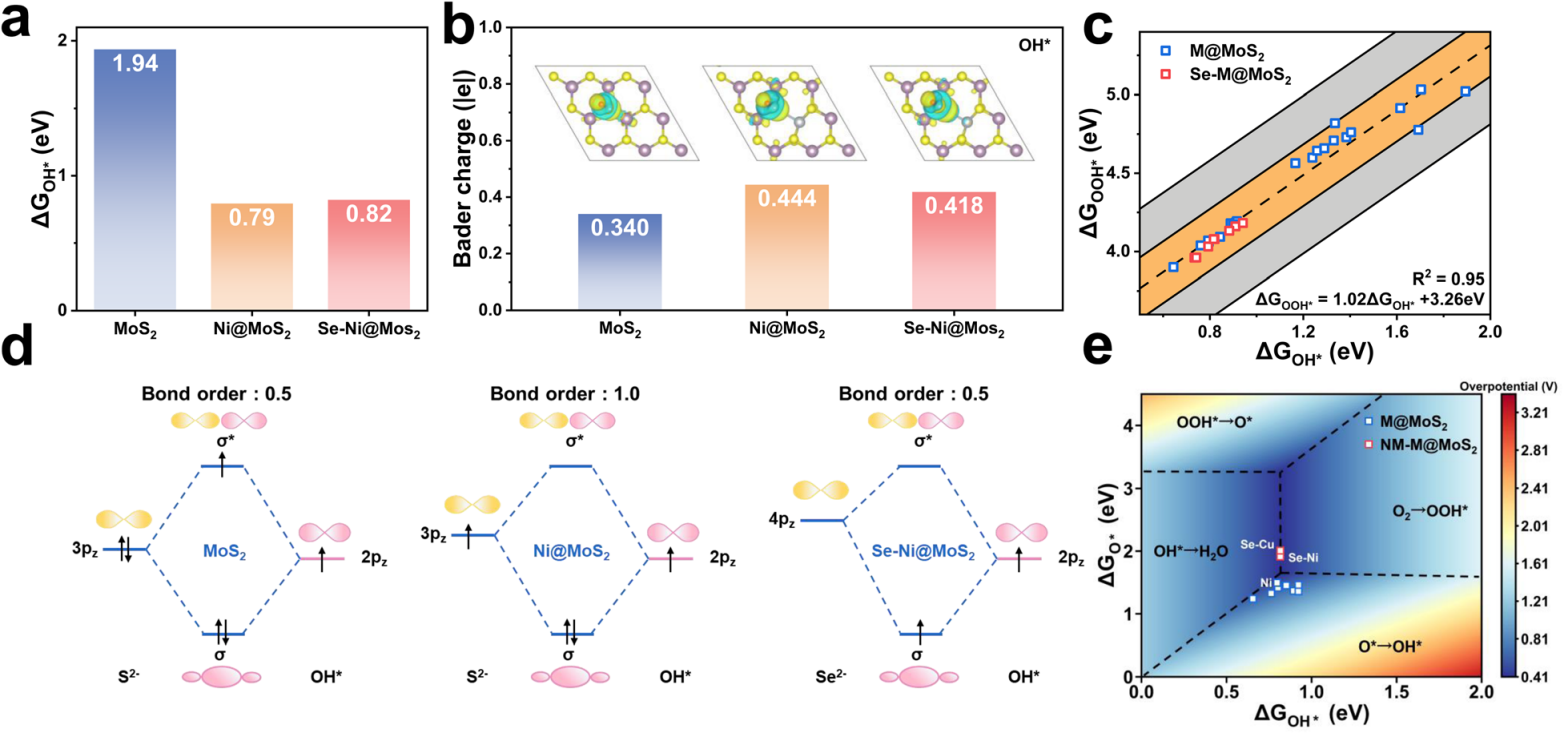
(a) The adsorption energies of OH*, and (b) the charge transfer values from S/Se to OH* for MoS_2_, Ni@MoS_2_ and Se–Ni@MoS_2_, respectively. (c) The scaling relationship between Δ*G*_OH*_ and Δ*G*_OOH*_ for different reaction sites in M@MoS_2_ and NM–M@MoS_2_. (d) The energy level splitting diagrams of MoS_2_, Ni@MoS_2_ and Se–Ni@MoS_2_, respectively. (e) 2D contour map of calculated Gibbs free energies of Δ*G*_OH*_ and Δ*G*_O*_ on the catalyst surface.

As presented in Fig. 7a, the adsorption energies of OH* and MoS_2_, Ni@MoS_2_ and Se–Ni@MoS_2_ are 1.94, 0.79, and 0.82 eV, respectively. Combined with Fig. 6d, although the p-band center of Se is close to the Fermi level, which enhances the catalytic performance, it also weakens the adsorption of OH*. It is worth noting that these results seem to be contrary to the previous discussion. Therefore, the electronic structures of OH* on the electrocatalysts are further analyzed, and the corresponding charge transfer between the active site and OH* is presented in Fig. 7b. Following Se doping, the adsorption capacity of the active site for OH* decreases, as evidenced by a reduction in the Bader charge from 0.444 |e| to 0.418 |e|. Fig. 7d illustrates the energy level diagram formed by the p orbitals of the active site and the OH* intermediate, *e.g.*, σ (S 3p–2p OH*), σ* (S 3p–2p OH*), σ (Se 4p–2p OH*), σ* (Se 4p–2p OH*). The bond order *b* is calculated to deepen the understanding and is expressed as follows11
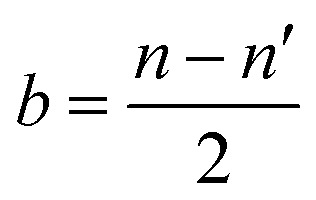
*n* and *n*′ represent the number of electrons in bonding and antibonding orbitals, respectively. The bonding and antibonding orbitals formed between metals and intermediates undergo the corresponding changes, *e.g.*, σ and σ*, thereby influencing the catalytic performance. Fig. 6e demonstrates that the introduction of dopant atoms leads to a decrease in the number of electrons at the active site, *i.e.*, S and Se. Therefore, the electron density in the σ* antibonding orbital decreases upon Ni-doping, thus enhance the bonding of S–OH*. Nevertheless, further decrease in the electron density, the bonding orbital is also reduced, thereby decrease the adsorption. This results in a trend of adsorption strength that first increases and then decreases from MoS_2_ to Ni@MoS_2_ to Se–Ni@MoS_2_, thus regulating the catalytic activity for the ORR. These findings suggest that, in accordance with the Sabatier principle, the p-band center of chalcogen-based active sites should reside at an optimal energy level to ensure moderate adsorption of oxygen intermediates. This balance prevents both excessively strong binding, which would impede reaction kinetics, and overly weak binding, which could cause premature desorption of intermediates.

Fig. 7c illustrates the relationship between Δ*G*_OH*_ and Δ*G*_OOH*_ of the constructed active centers. Notably, Cr@MoS_2_ and W@MoS_2_ are excluded due to their poor adsorption of intermediates. The Gibbs free energies of adsorbed OH* and OOH* follow a linear relationship, expressed as Δ*G*_OOH*_ = 1.02 Δ*G*_OH*_ + 3.26 eV, with a coefficient of determination *R*^2^ = 0.95, as shown by the black dashed line. In addition, the orange and gray shaded regions represent deviations from the fitted black line, with values of ±0.2 eV and ±0.5 eV, respectively. All active sites are fall within the yellow shaded region, indicating that the derived equation is reasonable and consistent with the catalyst model. Based on the relationship, the Gibbs free energy for ORR can be expressed by eqn (12)–(15):

12 Δ*G*_1_ = 4.92 − (1.02Δ*G*_OH*_ + 3.26)

13 Δ*G*_2_ = (1.02Δ*G*_OH*_ + 3.26) − Δ*G*_O*_

14 Δ*G*_3_ = Δ*G*_O*_ − Δ*G*_OH*_

15 Δ*G*_4_ = Δ*G*_OH*_

Consequently, the ORR performance can be evaluated with two descriptors, *i.e.*, Δ*G*_O*_ and Δ*G*_OH*_. To further screen the potential catalysts in various models, Fig. 7e further presents a two-dimensional volcano plot, which utilizes these independent descriptors. This methodology has been validated in numerous prior studies as effective predictors of the theoretical minimum overpotential for specific electrocatalytic models. As indicated by the black solid lines, the contour plot is divided into four distinct regions, each corresponding to a different PDS in the ORR process, *i.e.*, PDS1, PDS2, PDS3, and PDS4. It reveals that for MoS_2_ doped with d^7^–d^9^ transition metals, the predominant PDS is the Δ*G*_3_ step. In contrast, for Se–Ni@MoS_2_ and Se–Cu@MoS_2_, the primary PDS shifts to the Δ*G*_4_ step. The minimum theoretical overpotential can be derived from the condition Δ*G*_1_ = Δ*G*_4_ in the 2D volcano plot, which is 0.41 V.^52^ In conclusion, Se–Ni@MoS_2_ and Se–Cu@MoS_2_ exhibit the lowest overpotentials among the investigated systems, highlighting their superior catalytic activity. These findings underscore the significant potential of MoS_2_ as an ORR electrocatalyst.

## 4 Conclusions

The incorporation of specific heteroatoms enables precise regulation of the electronic structure and active sites of MoS_2_, thereby markedly enhancing its ORR catalytic performance. Nevertheless, the broad diversity of dopants has led to conflicting interpretations of the underlying mechanisms, leaving a significant knowledge gap in elucidating the structure–activity relationships that are critical for the rational design of high-performance MoS_2_-based ORR electrocatalysts. Accordingly, a total of 64 MoS_2_-based electrocatalysts, comprising transition metals from the 3d, 4d, and 5d series, together with selected non-metal dopants, are systematically examined to elucidate the underlying modulation mechanisms. This theoretical study demonstrates that the doped transition metals are classified into three categories according to their outermost d-electron count based on the calculated overpotentials and lattice distortions, *i.e.*, d^1^–d^3^, d^4^–d^6^, and d^7^–d^9^. In addition, the upward shift of the p-band center constitutes the critical factor underpinning the enhanced ORR electrocatalytic activity of MoS_2_; however, the underlying causes of this shift vary across different systems. For d^1^–d^3^, no structural distortion is observed, and the elevated sulfur p-band center is solely attributed to the d–p hybridization effect induced by metal doping. This upward shift in the p-band center leads to a reduction in ORR overpotential compared to pristine MoS_2_, with values ranging approximately from 0.87 to 1.07 V. In contrast, d^7^–d^9^ dopants induce the Jahn–Teller effect, breaking the orbital degeneracy between d_*xz*_/d_*yz*_ and d_*x*^2^−*y*^2^_/d_*xy*_, and leading to lattice distortion. The electron rearrangement at the metal center reduces charge transfer, thereby lowering the electron occupancy of the sulfur atom, upshifting its p-band center, and enhancing ORR performance by decreasing the overpotential to 0.53–0.74 V. Building on this, dual doping of NM–M@MoS_2_ with non-metal atoms is further explored, revealing that the incorporation of Se atoms yields the most significant reduction in overpotential, decreasing that of Se–Ni@MoS_2_ to 0.41 V. In summary, the enhancement mechanisms of MoS_2_ through substitutional doping can be broadly categorized into two types: d–p hybridization and the Jahn–Teller effect. These findings provide new theoretical insights for guiding the rational design of highly efficient MoS_2_-based ORR electrocatalysts. Furthermore, by bridging atomic-scale electronic structure modulation with macroscopic catalytic performance, this work establishes a robust theoretical framework that can guide the experimental synthesis and rational optimization of MoS_2_-based catalysts for practical deployment in fuel cells and metal-air battery systems.

## Author contributions

J.-C. Chen and M.-J. Pei performed the calculations and contributed to the manuscript preparation. Y. Liu and G.-Q. Luo were responsible for conceptualization, data curation, formal analysis, methodology, and funding acquisition. J. Zhang contributed to conceptualization, review and editing, and project administration. The other authors provided guidance and advice for this work. All authors have read and approved the final version of the manuscript.

## Conflicts of interest

The authors declare no conflict of interest.

## Supplementary Material

SC-OLF-D5SC07227A-s001

## Data Availability

The data that support the findings of this study are available from the corresponding author upon reasonable request. Supplementary information (SI) is available. See DOI: https://doi.org/10.1039/d5sc07227a.
