# Supplementary material for: New theoretical insights into doping-induced enhancement of ORR activity in molybdenum disulfide: d–p hybridization or the Jahn–Teller effect?
Source: Chem Sci. 2026 Jan 2;17(7):3758–74. doi: 10.1039/d5sc07227a (PMC12758488; doi:10.1039/d5sc07227a)
Supplement: SC-017-D5SC07227A-s001 [file SC-017-D5SC07227A-s001.pdf]

Supplementary Information (SI) for Chemical Science.
This journal is © The Royal Society of Chemistry 2025

New Theoretical Insights into Doping - Induced Enhancement of ORR Activity in Molybdenum Disulfide: d - p Hybridization or the Jahn - Teller Effect?

Jia-Cheng Chen, †, ‡ Mao-Jun Pei, †, ‡ Wen-Bei Yu, † Xiang Gao, † Qing Zeng, † Jia-Ming Xu, † Wei Yan, † Yao Liu, *, † Guo-Qiang Luo, *, ¶ and Jiujun Zhang*, †

† College of Materials Science & Engineering, Fuzhou University, 350108 Fuzhou, Fujian, China.

‡ J.-C. Chen and M.-J. Pei contributed equally to this paper
¶ State Key Lab of Advanced Technology for Materials Synthesis and Processing, Wuhan University of Technology, Wuhan, 430100, PR China

E-mail: yaoliu@fzu.edu.cn; luogq@whut.edu.cn; jiujun.zhang@fzu.edu.cn

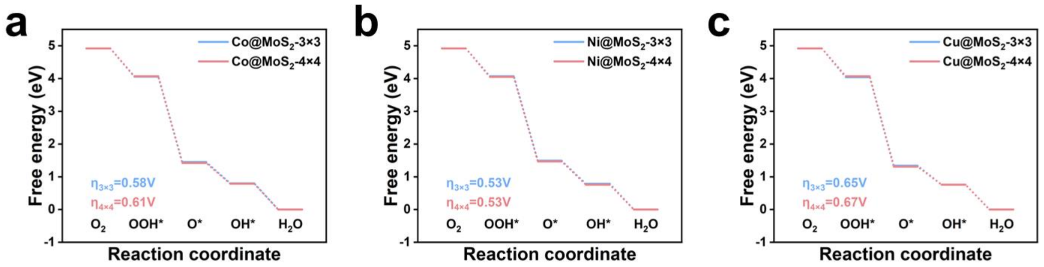

Fig. S1 Comparison of ORR performance for different cell sizes (a) Co@MoS_2, (b) Ni@MoS_2, and (c) Cu@MoS_2.

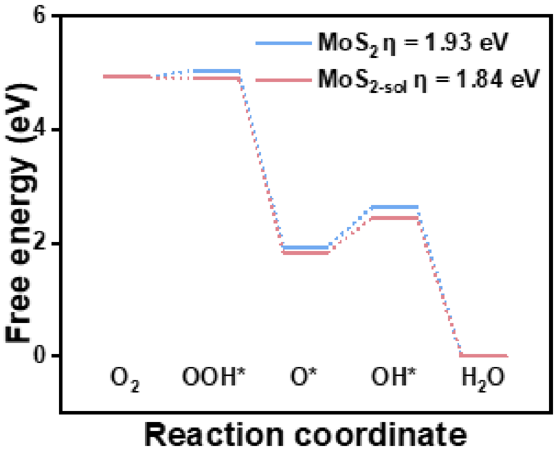

Fig. S2 Free-energy diagrams of MoS_2 and $MoS_{2\text{-sol}}$.

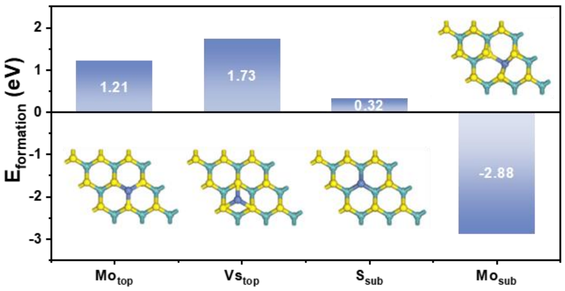

Fig. S3 Formation energies of different doping configurations. Mo_{top}: Ni doped above the Mo site; Vs_{top}: Ni doped into the surface hollow site; S_{sub}: Ni substituting an S atom; Mo_{sub}: Ni substituting a Mo atom.

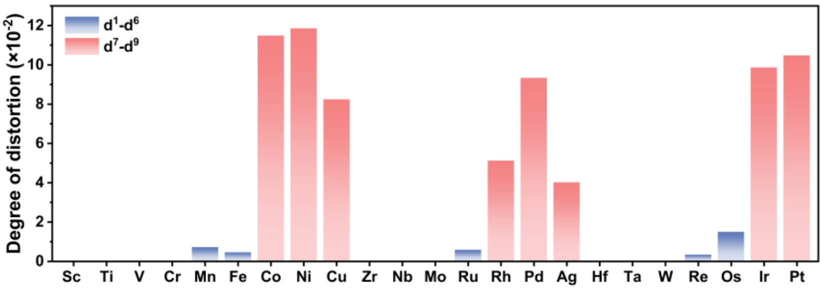

Fig. S4 Degree of structural distortion in MoS_2 doped with different metal species.

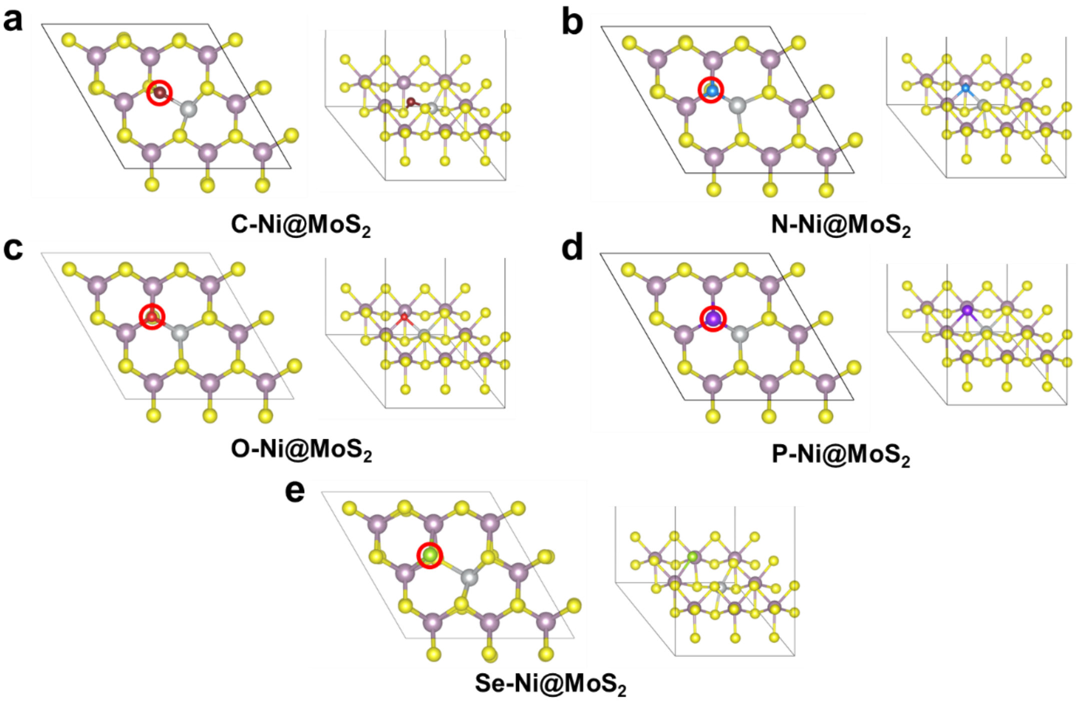

Fig. S5 Optimized structures of Ni-doped MoS_2 with different heteroatom modifications, shown in side and enlarged views: (a) C-Ni@MoS_2, (b) N-Ni@MoS_2, (c) O-Ni@MoS_2, (d) P-Ni@MoS_2, and (e) Se-Ni@MoS_2.

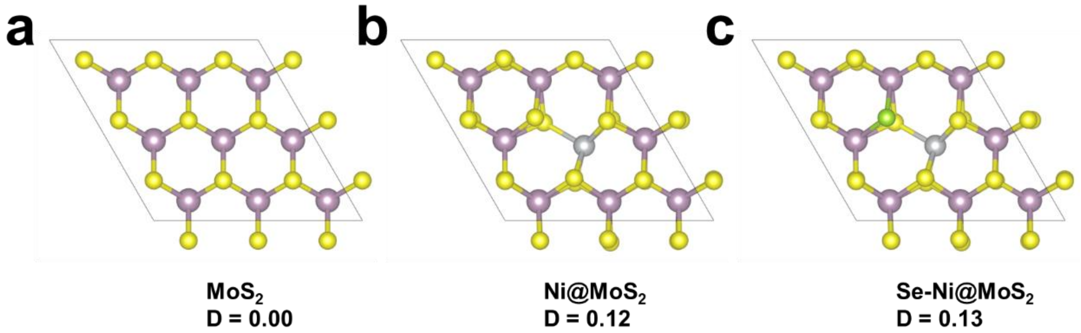

Fig. S6 Optimized structures and corresponding distortion degrees of (a) pristine MoS_2, (b) Ni@MoS_2, and (c) Se-Ni@MoS_2.

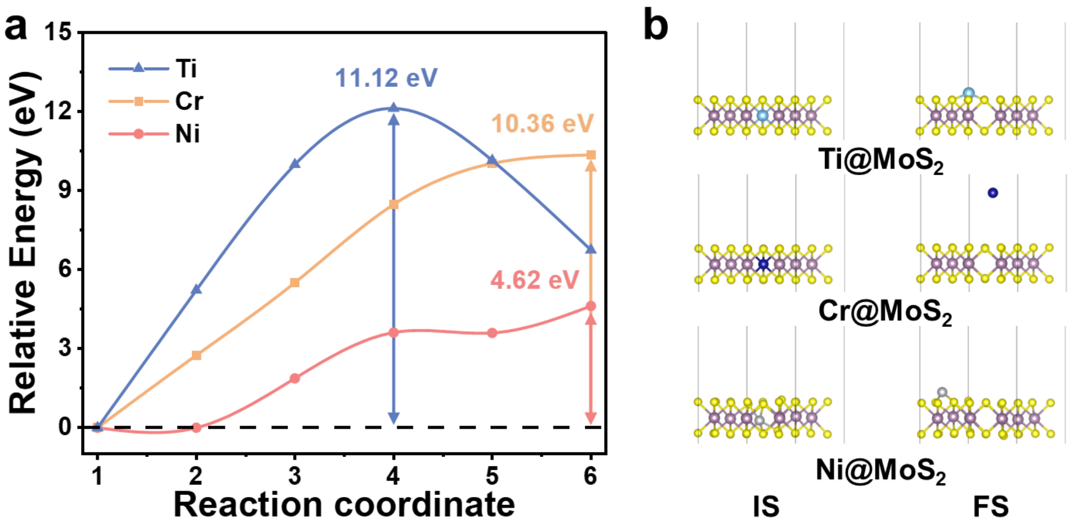

Fig. S7 (a) Calculated migration energy barriers for Ti, Cr, and Ni dopants in MoS_2. (b) Initial (IS) and final (FS) configurations corresponding to dopant migration pathways.

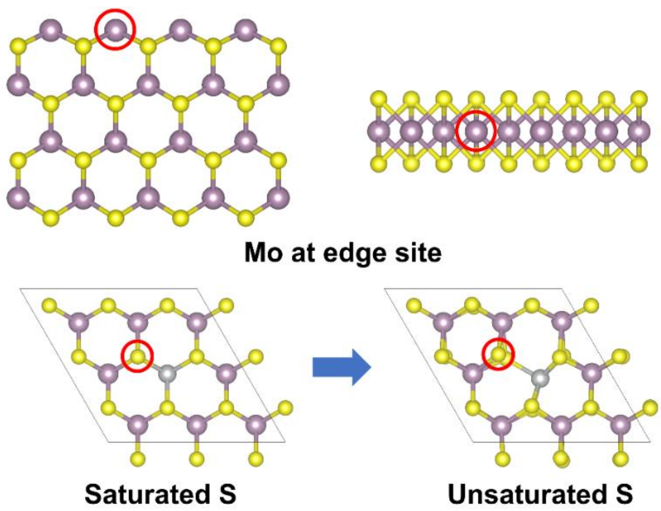

Fig. S8 Schematic illustration of edge Mo sites and surface S sites on MoS_2.

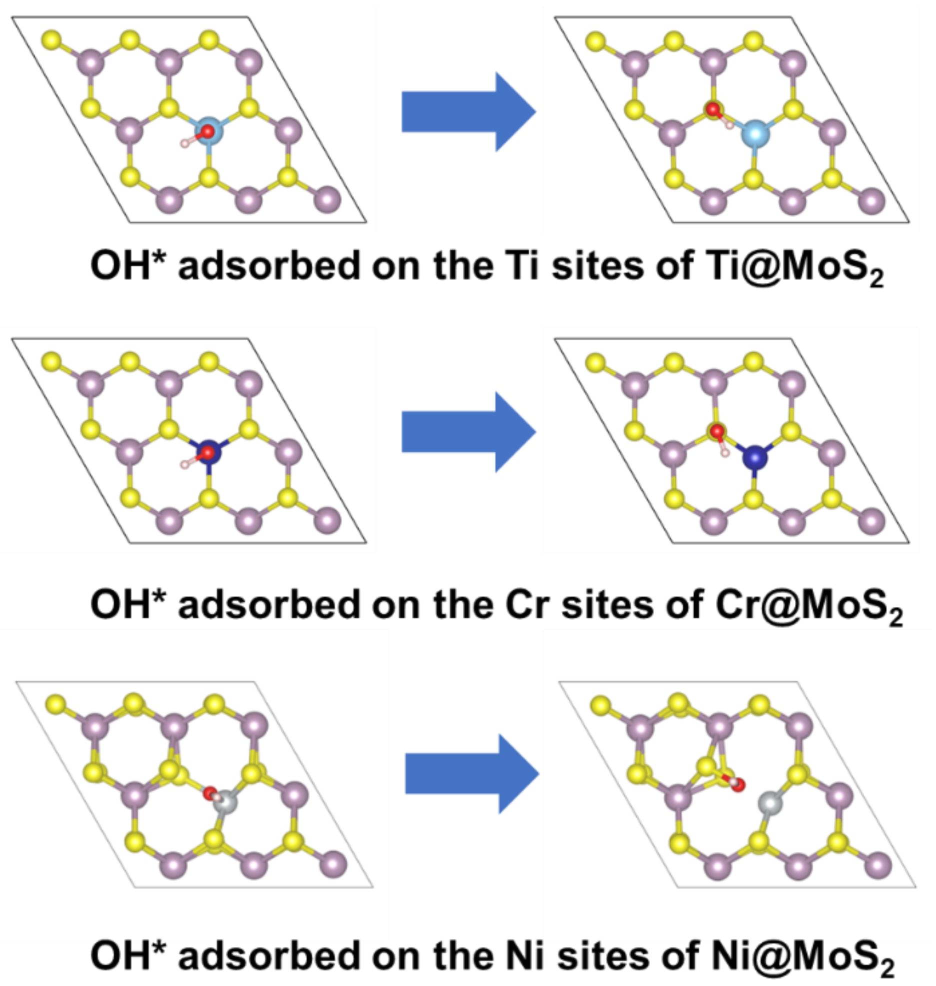

Fig. S9 Structural evolution before and after OH* adsorption on the M site of Ti@MoS_2, Cr@MoS_2, and Ni@MoS_2.

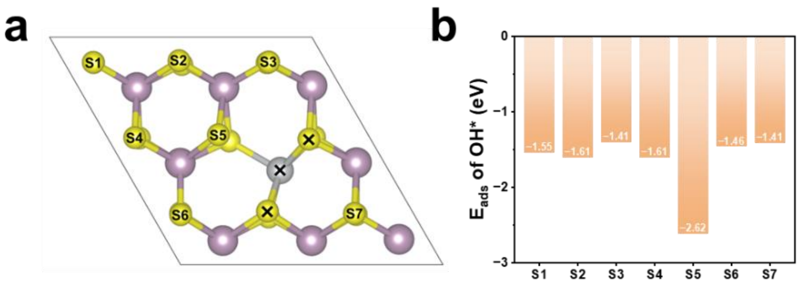

Fig. S10 (a) Schematic illustration of the different adsorption sites on Ni@MoS_2. (b) OH* adsorption energies at the various surface S sites of Ni@MoS_2.

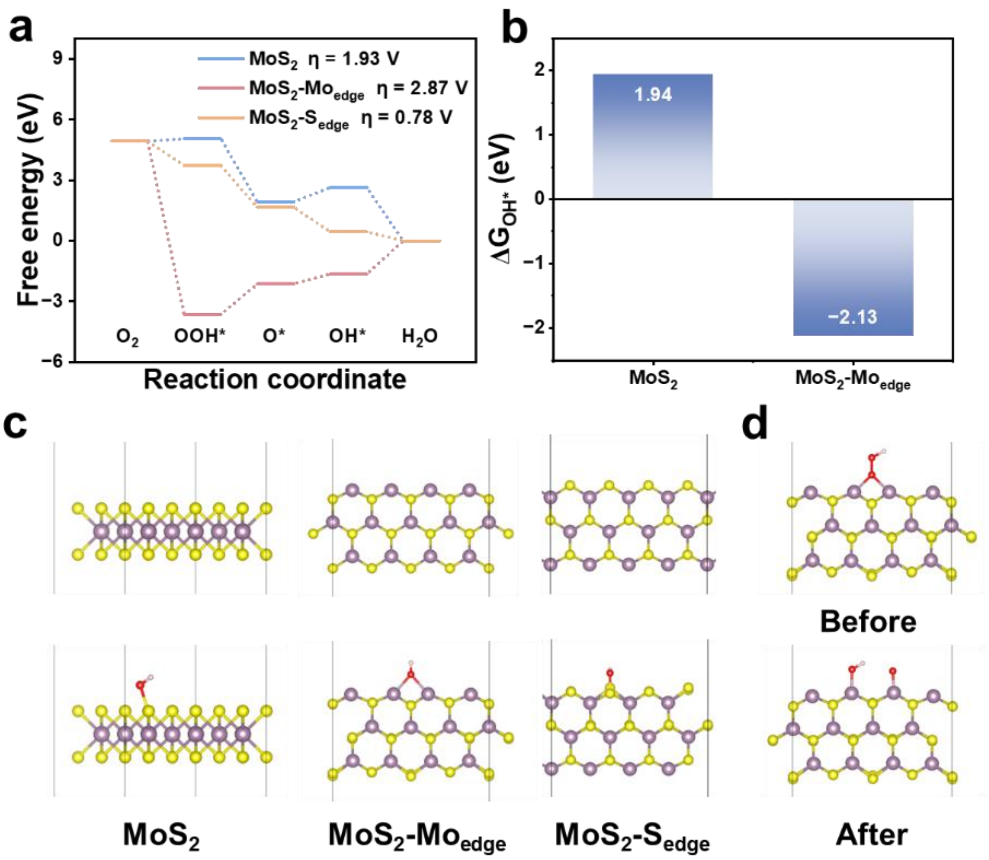

Fig. S11 (a) Free-energy diagrams of MoS_2, MoS_2-S_{edge} and MoS_2-Mo_{edge}. (b) Gibbs free energy of OH* adsorption on MoS_2 and MoS_2-Mo_{edge}. (c) Slab structures and corresponding OH* adsorption configurations on MoS_2, MoS_2-S_{edge} and MoS_2-Mo_{edge}. (d) Initial and optimized OOH* adsorption configurations on MoS_2-Mo_{edge}.

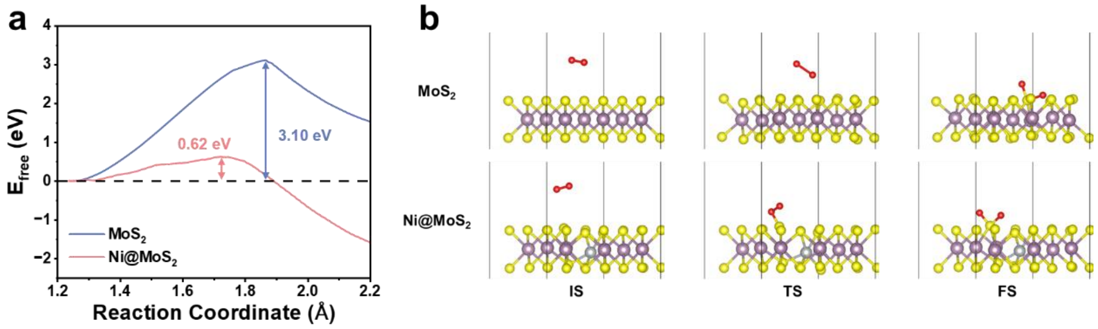

Fig. S12 (a) Calculated free-energy barriers for O_2 dissociation on MoS_2 and $Ni@MoS_2$. (b) Corresponding initial (IS), transition (TS), and final (FS) configurations of O_2 dissociation on each surface.

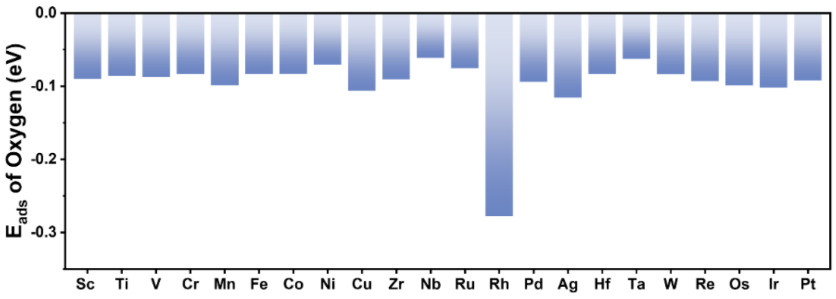

Fig. S13 Oxygen adsorption energies on MoS_2 doped with different metal species.

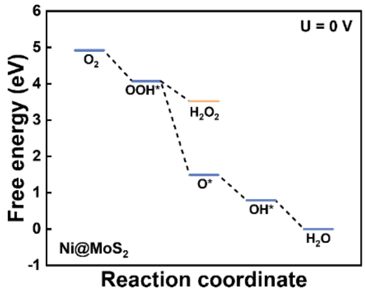

Fig. S14 Free-energy diagram of Ni@MoS_2 at zero applied potential.

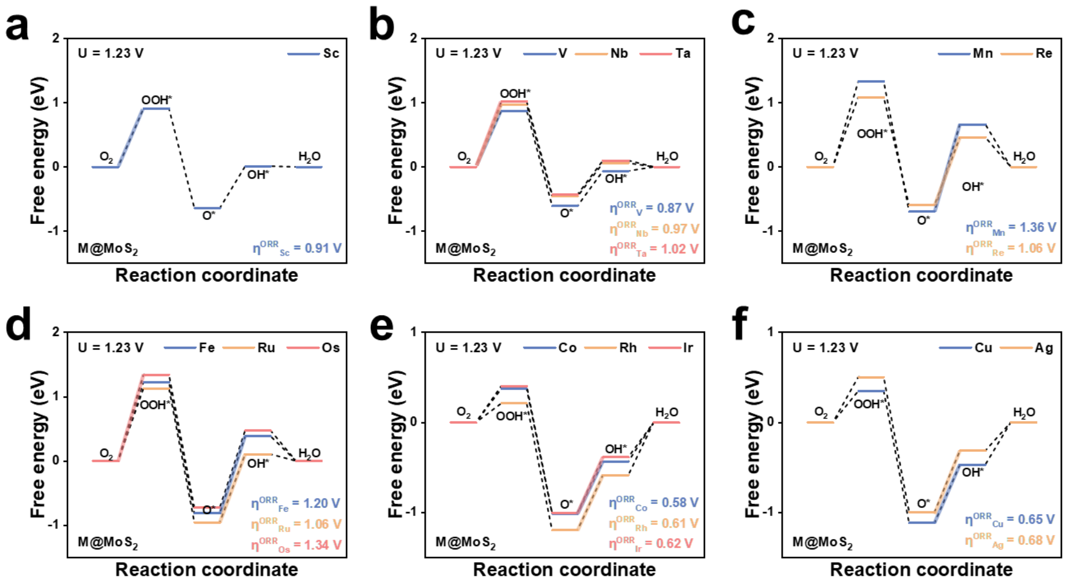

Fig. S15 Free energy diagrams for the ORR at U = 1.23 V on M@MoS_2 doped with different transition metals: (a) Sc, (b) V, Nb, Ta, (c) Mn, Re, (d) Fe, Ru, Os, (e) Co, Rh, Ir, and (f) Cu, Ag.

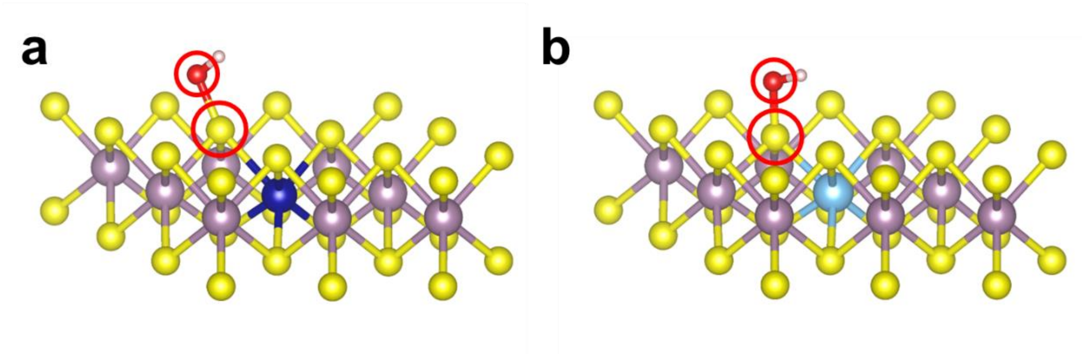

Fig. S16 The selected atoms in figures 4b and 4c.

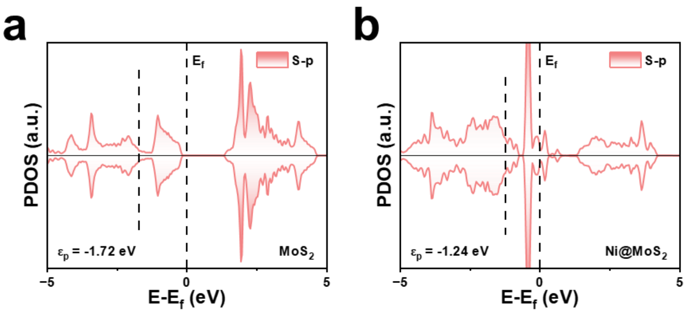

Fig. S17 PDOS of S atoms in MoS_2 and Ni@MoS_2.

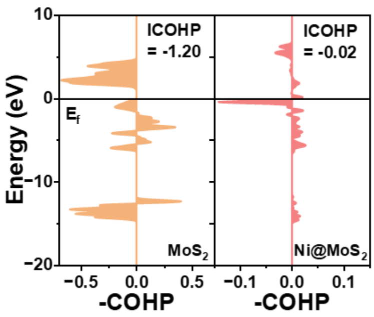

Fig. S18 COHP analysis of MoS_2 and Ni@MoS_2.

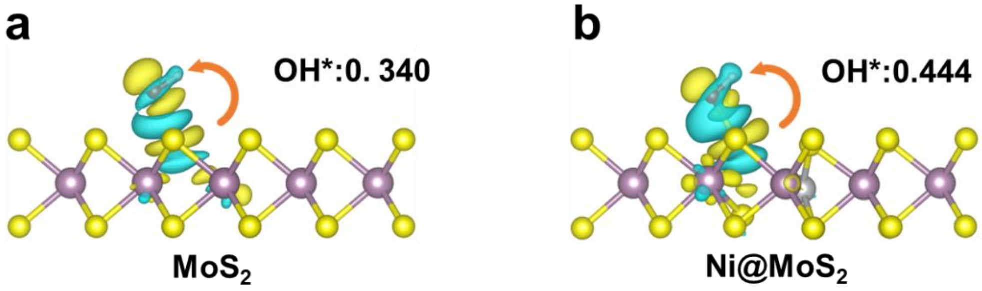

Fig. S19 Differential charge density and Bader charge analyses of (a) MoS_2 and (b) Ni@MoS_2 after OH* adsorption.

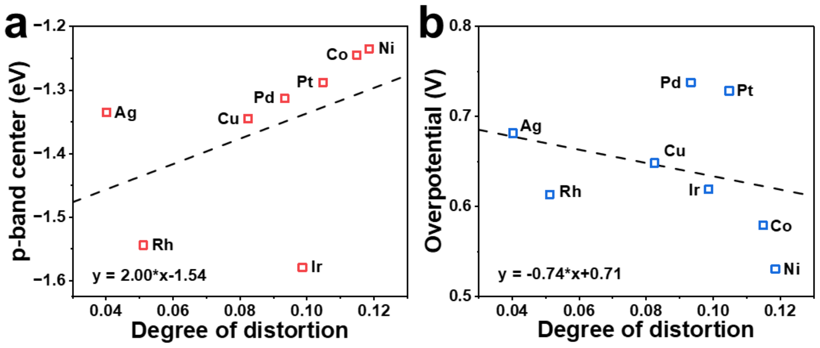

Fig. S20 Correlation between the degree of structural distortion and (a) the sulfur p-band center (ε_p) and (b) the ORR overpotential (η) for d^7-d^9 transition metal dopants.

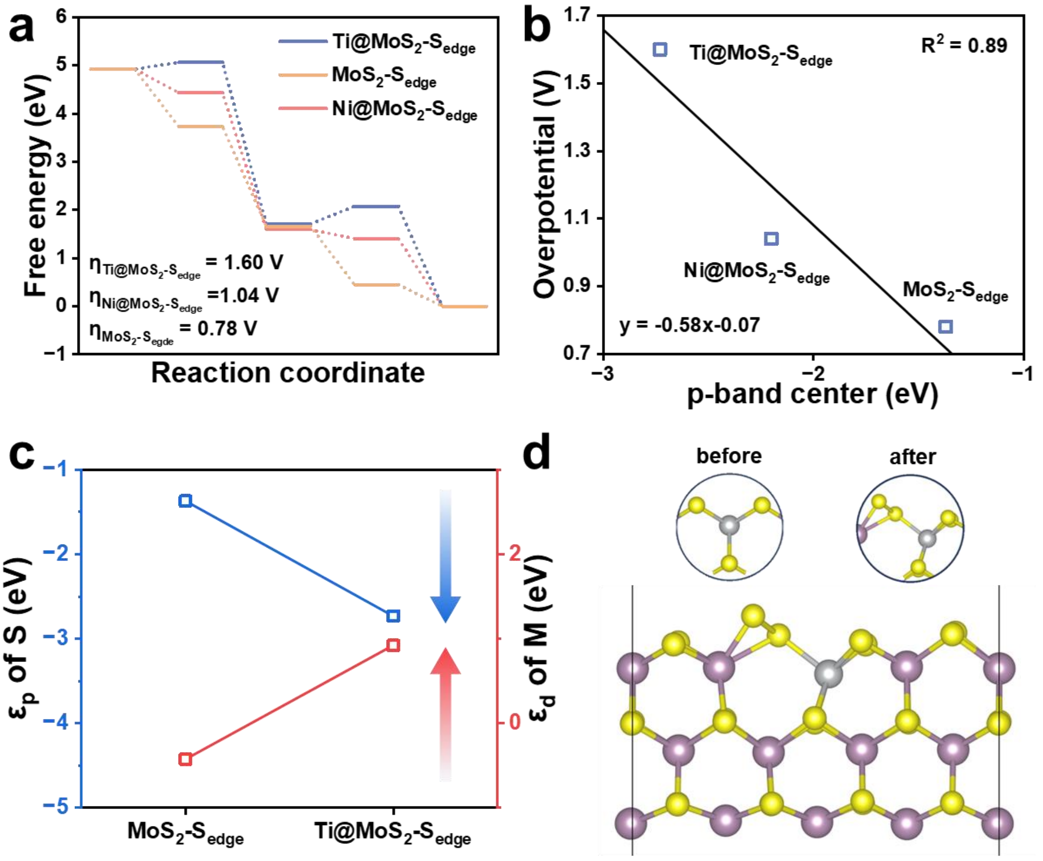

Fig. S21 (a) Free energy diagrams of MoS_2-S_{edge}, Ti@MoS_2-S_{edge} and Ni@MoS_2-S_{edge}. (b) Scaling relationship between η^{ORR} and ε_p for MoS_2-S_{edge}, Ti@MoS_2-S_{edge} and Ni@MoS_2-S_{edge}. (c) ε_d of M and ε_p of S for MoS_2-S_{edge} and Ti@MoS_2-S_{edge}. (d) Structures of Ni@MoS_2-S_{edge} before and after distortion.

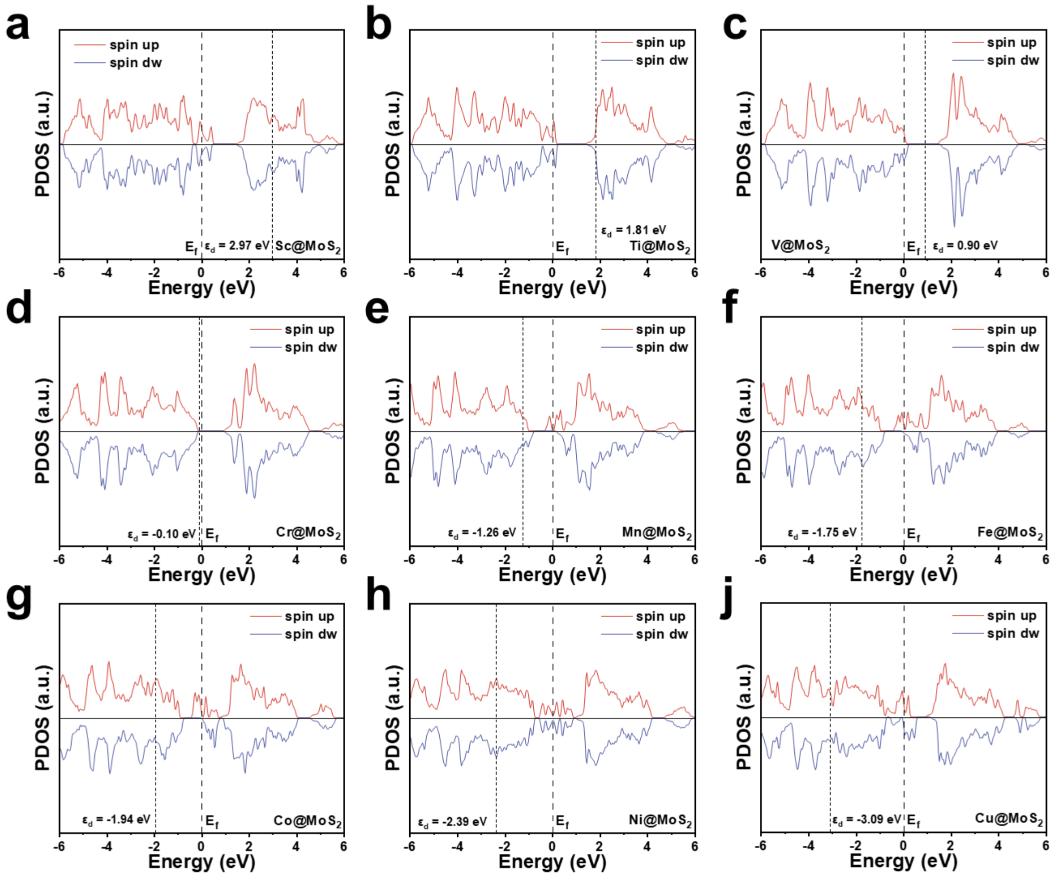

Fig. S22 PDOS for transition metal doped MoS_2: (a) Sc, (b) Ti, (c) V, (d) Cr, (e) Mn, (f) Fe, (g) Co, (h) Ni, and (j) Cu. ε_d indicates the d-band center.

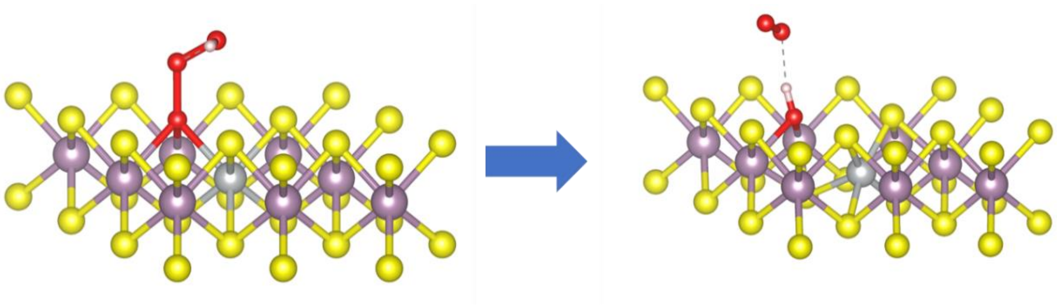

Fig. S23 Structural evolution of O-Ni@MoS_2 before and after optimization with OOH* adsorption.

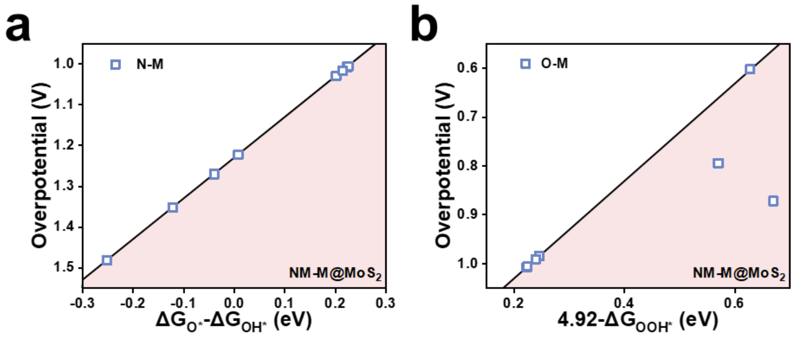

Fig. S24 Volcano plots of N-M@MoS_2 and O-M@MoS_2.

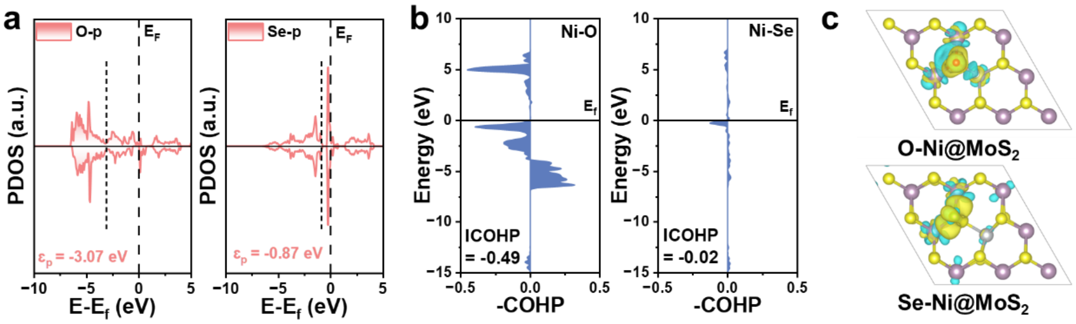

Fig. S25 (a) PDOS of O-p and Se-p orbitals in O-Ni@MoS_2 and Se-Ni@MoS_2. (b) -COHP for Ni-O and Ni-Se interactions. (c) Differential charge density plots of O-Ni@MoS_2 and Se-Ni@MoS_2, showing interfacial charge redistribution.

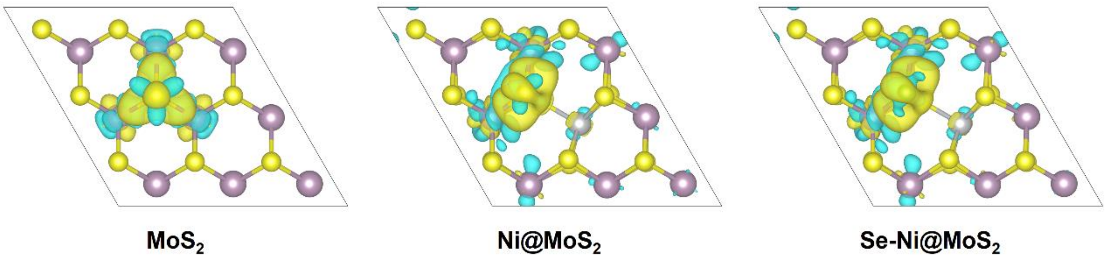

Fig. S26 Charge density difference plots of MoS_2, Ni@MoS_2, and Ni-Se@MoS_2.

Table S1. Formation energies and dissolution potentials of $M@MoS_2$ and NM-$M@MoS_2$

Samples	E_{for} (eV)	U_{diss} (V)	Samples	E_{for} (eV)	U_{diss} (V)
$Sc@MoS_2$	-6.79	0.18	$Y@MoS_2$	-6.12	-0.33
$Ti@MoS_2$	-7.59	2.16	$Zr@MoS_2$	-7.72	0.48
$V@MoS_2$	-7.20	2.42	$Nb@MoS_2$	-8.02	1.57
$Cr@MoS_2$	-6.55	2.37	$Ru@MoS_2$	-4.44	2.68
$Mn@MoS_2$	-5.43	1.52	$Rh@MoS_2$	-3.41	2.30
$Fe@MoS_2$	-4.27	1.68	$Pd@MoS_2$	-2.81	2.36
$Co@MoS_2$	-3.56	1.50	$Ag@MoS_2$	-0.77	1.57
$Ni@MoS_2$	-2.88	1.18			
$Cu@MoS_2$	-1.52	1.10			
$Hf@MoS_2$	-7.69	0.37	C-$Co@MoS_2$	-1.74	0.59
$Ta@MoS_2$	-7.80	2.00	C-$Ni@MoS_2$	-2.14	0.81
$W@MoS_2$	-7.39	2.56	C-$Cu@MoS_2$	-0.51	0.60
$Re@MoS_2$	-5.60	2.17	C-$Rh@MoS_2$	-1.94	1.57
$Os@MoS_2$	-4.38	1.39	C-$Pd@MoS_2$	-0.70	1.30
$Ir@MoS_2$	-3.41	2.30	C-$Ag@MoS_2$	1.12	-0.32
$Pt@MoS_2$	-3.05	2.70	C-$Ir@MoS_2$	-2.05	1.84
$Hf@MoS_2$	0.09	1.47	C-$Pt@MoS_2$	-0.94	1.65
N-$Co@MoS_2$	-2.79	1.11	O-$Co@MoS_2$	-5.41	2.43
N-$Ni@MoS_2$	-1.59	0.54	O-$Ni@MoS_2$	-4.40	1.94
N-$Cu@MoS_2$	-0.58	0.63	O-$Cu@MoS_2$	-3.78	2.23
N-$Rh@MoS_2$	-3.01	2.10	O-$Rh@MoS_2$	-5.92	3.56
N-$Pd@MoS_2$	-1.95	1.92	O-$Pd@MoS_2$	-5.08	3.49
N-$Ag@MoS_2$	0.04	0.76	O-$Ag@MoS_2$	-3.09	3.89
N-$Ir@MoS_2$	-3.02	2.17	O-$Ir@MoS_2$	-5.69	3.06
N-$Pt@MoS_2$	-1.92	2.14	O-$Pt@MoS_2$	-5.32	3.84

Table S1. continued

Samples	E_{for} (eV)	U_{diss} (V)	Samples	E_{for} (eV)	U_{diss} (V)
P-Co@MoS_2	-3.78	1.61	Se-Co@MoS_2	-4.26	1.85
P-Ni@MoS_2	-2.63	1.05	Se-Ni@MoS_2	-3.56	1.52
P-Cu@MoS_2	-1.43	1.06	Se-Cu@MoS_2	-2.23	1.46
P-Rh@MoS_2	-4.07	2.63	Se-Rh@MoS_2	-4.25	2.73
P-Pd@MoS_2	-2.86	2.38	Se-Pd@MoS_2	-3.47	2.68
P-Ag@MoS_2	-1.00	1.80	Se-Ag@MoS_2	-1.45	2.25
P-Ir@MoS_2	-4.10	2.53	Se-Ir@MoS_2	-4.09	2.52
P-Pt@MoS_2	-3.08	2.72	Se-Pt@MoS_2	-3.70	3.03

Table S2. Reaction free energies ΔGi of intermediate steps (U = 0V) and the overpotentials at metal active sites of all M@MoS_2 samples.

Samples	ΔG_1 (eV)	ΔG_2 (eV)	ΔG_3 (eV)	ΔG_4 (eV)	η(V)
Sc@MoS_2	**0.32**	2.78	0.58	1.24	0.91
Ti@MoS_2	**0.28**	2.80	0.58	1.26	0.95
V@MoS_2	**0.36**	2.71	0.69	1.17	0.87
Cr@MoS_2	-0.12	3.26	**-0.54**	2.32	1.77
Mn@MoS_2	-0.10	3.26	**-0.13**	1.89	1.36
Fe@MoS_2	0.00	3.27	**0.03**	1.61	1.20
Co@MoS_2	0.86	2.61	**0.65**	0.80	0.58
Ni@MoS_2	0.85	2.58	**0.70**	0.79	0.53
Cu@MoS_2	0.88	2.70	**0.58**	0.76	0.65
Zr@MoS_2	**0.19**	2.81	0.54	1.38	1.04
Nb@MoS_2	**0.26**	2.66	0.71	1.29	0.97
MoS_2	-0.13	3.11	**-0.70**	2.64	1.93
Ru@MoS_2	0.10	3.31	**0.17**	1.34	1.06
Rh@MoS_2	1.01	2.64	**0.62**	0.65	0.61
Pd@MoS_2	0.73	2.78	**0.49**	0.91	0.74
Ag@MoS_2	0.90	2.73	**0.55**	0.74	0.68
Hf@MoS_2	**0.16**	2.83	0.53	1.40	1.07
Ta@MoS_2	**0.21**	2.68	0.70	1.33	1.02
W@MoS_2	-0.48	3.41	**-0.64**	2.64	1.87
Re@MoS_2	0.14	2.92	**0.17**	1.69	1.06
Os@MoS_2	**-0.11**	3.30	0.03	1.70	1.34
Ir@MoS_2	1.00	2.64	**0.61**	0.67	0.62
Pt@MoS_2	0.75	2.77	**0.50**	0.90	0.73

Table S3. The p-band center and η of representative catalysts

Samples	ε_p (eV)	η(V)
Ti@MoS_2	-1.49	0.95
Zr@MoS_2	-1.51	1.04
Hf@MoS_2	-1.63	1.07
Cr@MoS_2	-1.76	1.77
MoS_2	-1.72	1.92
W@MoS_2	-1.81	1.87
Ni@MoS_2	-1.24	0.53
Pd@MoS_2	-1.31	0.74
Pt@MoS_2	-1.29	0.73

Table S4. Reaction free energies ΔGi of intermediate steps (U = 0V) and the overpotentials at metal active sites of all NM-M@MoS_2 samples.

Samples	ΔG_1 (eV)	ΔG_2 (eV)	ΔG_3 (eV)	ΔG_4 (eV)	η(V)
C-Co@MoS_2	3.02	3.36	-0.22	**-1.24**	2.47
C-Ni@MoS_2	1.70	3.37	**-0.32**	0.17	1.55
C-Cu@MoS_2	4.82	0.63	-0.10	**-0.43**	1.66
C-Rh@MoS_2	3.04	3.27	-0.07	**-1.32**	2.55
C-Pd@MoS_2	5.61	0.71	0.08	**-1.48**	2.71
C-Ag@MoS_2	5.76	0.82	-0.17	**-1.49**	2.72
C-Ir@MoS_2	2.88	3.17	0.07	**-1.21**	2.44
C-Pt@MoS_2	5.50	0.73	0.15	**-1.46**	2.69
N-Co@MoS_2	0.75	3.08	**-0.04**	1.14	1.27
N-Ni@MoS_2	0.91	3.36	**-0.25**	0.90	1.48
N-Cu@MoS_2	1.12	2.86	**0.20**	0.74	1.03
N-Rh@MoS_2	0.49	3.19	**0.01**	1.23	1.22
N-Pd@MoS_2	0.89	2.92	**0.22**	0.89	1.01
N-Ag@MoS_2	0.99	2.92	**0.23**	0.79	1.00
N-Ir@MoS_2	0.25	3.28	**-0.12**	1.51	1.35
N-Pt@MoS_2	1.25	2.83	**0.21**	0.62	1.02
O-Co@MoS_2	**0.63**	0.77	1.00	2.53	0.60
O-Ni@MoS_2	1.36	0.40	**0.25**	2.92	0.98
O-Cu@MoS_2	0.67	**0.36**	0.93	2.96	0.87
O-Rh@MoS_2	**0.22**	0.66	1.12	2.92	1.01
O-Pd@MoS_2	0.57	**0.44**	0.98	2.94	0.79
O-Ag@MoS_2	**0.22**	0.73	1.04	2.92	1.01
O-Ir@MoS_2	**0.25**	0.65	1.13	2.90	0.98
O-Pt@MoS_2	**0.24**	0.77	1.00	2.91	0.99
P-Co@MoS_2	1.80	3.15	0.36	**-0.39**	1.62
P-Ni@MoS_2	2.20	2.77	0.73	**-0.77**	2.00
P-Cu@MoS_2	1.96	3.07	0.43	**-0.54**	1.77
P-Rh@MoS_2	1.42	3.46	0.06	**-0.02**	1.25
P-Pd@MoS_2	1.67	3.24	0.26	**-0.25**	1.48
P-Ag@MoS_2	1.45	3.50	0.01	**-0.04**	1.27
P-Ir@MoS_2	3.11	1.72	**-0.04**	0.13	1.27
P-Pt@MoS_2	3.42	1.42	0.35	**-0.27**	1.50

Table S4. continued

Samples	ΔG_1 (eV)	ΔG_2 (eV)	ΔG_3 (eV)	ΔG_4 (eV)	η(V)
Se-Co@MoS_2	0.89	2.05	1.19	**0.79**	0.44
Se-Ni@MoS_2	0.84	2.16	1.10	**0.82**	0.41
Se-Cu@MoS_2	0.84	2.11	1.15	**0.82**	0.41
Se-Rh@MoS_2	0.96	2.05	1.17	**0.73**	0.50
Se-Pd@MoS_2	**0.76**	2.21	1.04	0.91	0.47
Se-Ag@MoS_2	**0.74**	2.13	1.11	0.94	0.49
Se-Ir@MoS_2	0.96	2.05	1.17	**0.74**	0.49
Se-Pt@MoS_2	**0.79**	2.21	1.04	0.88	0.44
